# Methylsiloxanes from Vehicle Emissions Detected in Aerosol Particles

**DOI:** 10.1021/acs.est.3c03797

**Published:** 2023-09-12

**Authors:** Peng Yao, Rupert Holzinger, Dušan Materić, Beatriz Sayuri Oyama, Maria de Fátima Andrade, Dipayan Paul, Haiyan Ni, Hanne Noto, Ru-Jin Huang, Ulrike Dusek

**Affiliations:** † Centre for Isotope Research (CIO), Energy and Sustainability Research Institute Groningen (ESRIG), University of Groningen, Groningen 9747 AG, The Netherlands; ‡ Institute for Marine and Atmospheric Research, IMAU, Utrecht University, Princetonplein 5, Utrecht 3584 CC, The Netherlands; § Institute of Astronomy, Geophysics and Atmospheric Sciences, University of São Paulo, São Paulo 05508-090, Brazil; ∥ State Key Laboratory of Loess and Quaternary Geology, Center for Excellence in Quaternary Science and Global Change, Key Laboratory of Aerosol Chemistry & Physics, Institute of Earth Environment, Chinese Academy of Sciences, Xi’an 710061, China; ⊥ Department of Analytical Chemistry, Helmholtz Centre for Environmental Research—UFZ, Permoserstrasse 15, 04318 Leipzig, Germany

**Keywords:** methylsiloxane, aerosol, vehicle emissions, fuel, lubricating oil

## Abstract

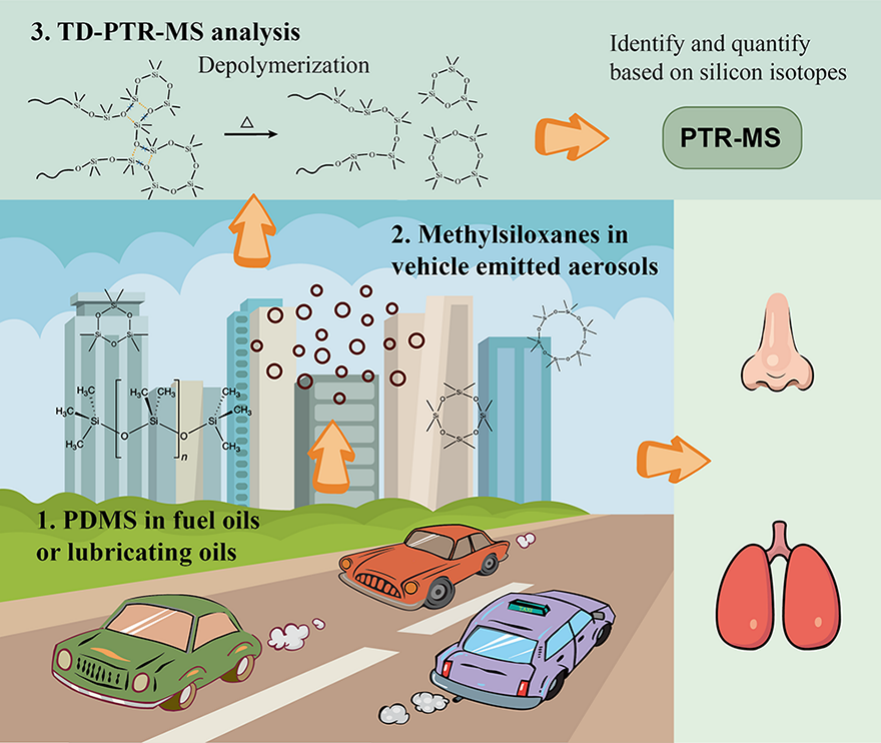

Methylsiloxanes have gained growing attention as emerging pollutants due to their toxicity to organisms. As man-made chemicals with no natural source, most research to date has focused on volatile methylsiloxanes from personal care or household products and industrial processes. Here, we show that methylsiloxanes can be found in primary aerosol particles emitted by vehicles based on aerosol samples collected in two tunnels in São Paulo, Brazil. The aerosol samples were analyzed with thermal desorption-proton transfer reaction-mass spectrometry (TD-PTR-MS), and methylsiloxanes were identified and quantified in the mass spectra based on the natural abundance of silicon isotopes. Various methylsiloxanes and derivatives were found in aerosol particles from both tunnels. The concentrations of methylsiloxanes and derivatives ranged 37.7–377 ng m^–3^, and the relative fractions in organic aerosols were 0.78–1.9%. The concentrations of methylsiloxanes exhibited a significant correlation with both unburned lubricating oils and organic aerosol mass. The emission factors of methylsiloxanes averaged 1.16 ± 0.59 mg kg^–1^ of burned fuel for light-duty vehicles and 1.53 ± 0.37 mg kg^–1^ for heavy-duty vehicles. Global annual emissions of methylsiloxanes in vehicle-emitted aerosols were estimated to range from 0.0035 to 0.0060 Tg, underscoring the significant yet largely unknown potential for health and climate impacts.

## Abstract 2

Considerable amounts of methylsiloxanes were identified and quantified in vehicle-emitted aerosols for the first time.

## 1 Introduction

Methylsiloxanes are oligomers or polymers composed of −Si(CH_3_)_2_–O– units.^1^ Among them, volatile methylsiloxanes (VMS) are small molecular oligomers with cyclic or linear forms, and poly(dimethylsiloxanes) (PDMS) are non-volatile polymers, a type of silicone oil. In the following description, the term methylsiloxanes refers to all kinds of methylsiloxane molecules, including small molecular VMS, large molecular PDMS, and molecules in between. VMS are commonly used in various cosmetics, household products,^2,3^ and industrial products.^1^ Due to the health effects, VMS have been studied intensively.^4,5^ VMS were found in the plasma of residents,^6,7^ and hydrolyzed products of methylsiloxanes were detected in the human metabolites.^8^ Exposure to VMS can cause liver, endometrial, and respiratory damage, endocrine disturbances, and adverse immune responses.^9−11^ However, the long-term toxicity and bioaccumulation of methylsiloxanes are still unclear and need further investigation.^10,12^

Considering the differences in disciplines, we clarify that air only refers to the gas phase, aerosol refers to the particulate matter in the air/atmosphere, and dust refers to deposited particulate matter on the ground. As an emerging class of pollutant with relatively high volatility, VMS have been found mainly in the gas phase, such as indoor air^13^ and outdoor air.^14−16^ VMS were also found in indoor dust samples,^17−20^ originating possibly from personal care products, rubber products, and electrical/electronic appliances.

On the other hand, methylsiloxanes in aerosols have not received much attention to date, and only a few studies have reported methylsiloxanes in indoor aerosols.^6,20−22^ Most studies hypothesize that the methylsiloxanes in the particle phase are mainly derived directly or indirectly from the gas phase. In short, VMS can be oxidized into low-volatile products, which then condense on particles.^23^ Some studies have found evidence to support gas-to-particle conversion in indoor environments, i.e., VMS in the indoor air were associated with VMS in indoor dust^19^ and indoor aerosols.^6^ Primary emissions of particle-phase methylsiloxanes were less investigated and only found in some special indoor environments, e.g., by heating silicone baking molds in an oven.^24,25^

However, our recent study revealed a substantial presence of methylsiloxanes in aerosol particles derived from ship emissions.^26^ This discovery suggests that engine combustion may be a potential and widespread source of methylsiloxanes, which has been overlooked thus far. Methylsiloxanes emitted by engine combustion likely have their origin in PDMS contained in fuel oils or lubricating oils, where they serve as antifoam additives.^27−32^ The high thermal resistance of PDMS implies that fragments and unburned PDMS can potentially be emitted by vehicle engines. Furthermore, the combustion of PDMS may result in smaller fragments, as well as oxidized and hydrolyzed products with various molecular sizes in the emitted gases and particles.

Using thermal desorption-proton transfer reaction-mass spectrometry (TD-PTR-MS) and an evaluation method based on the natural abundance of silicon isotopes as described in our recent study,^26^ the identification and quantification of methylsiloxanes in a complex mixture become possible. In this study, we applied the aforementioned method to previously measured data of aerosol samples from two tunnels in Brazil to identify and quantify methylsiloxanes from vehicle emissions. Additionally, emission factors of methylsiloxanes were determined, and global emissions were estimated.

## 2 Materials and Methods

### 2.1 PDMS Standards

To study the thermal desorption and depolymerization properties of PDMS standards with different molecular sizes, commercially available PDMS samples of different viscosities (5, 10, 20, 50, 100, 1000, 10,000 cSt at 25 °C, Sigma-Aldrich) were analyzed, referred to as PDMS5, PDMS10, PDMS20, PDMS50, PDMS100, PDMS1000, and PDMS10000. The viscosities of 5, 10, 20, 50, 100, 1000, and 10,000 cSt correspond to 8, 15, 25, 50, 80, 400, and 800 siloxane units, respectively, as shown in Table S5, which was adapted from Mojsiewicz-Pieńkowska et al.^33^ The PDMS standard samples were first dissolved in n-hexane (99%, Macron Fine Chemicals) and loaded onto the quartz filters (Whatman, QMA 1851-150, precleaned at 650 °C for 2 h). The solvent *n*-hexane was evaporated at 50 °C for 2 h. The PDMS samples were wrapped in an aluminum foil (precleaned at 550 °C for 2 h) and stored inside plastic bags in a freezer at −20 °C. The thermal resistance of PDMS of various sizes was investigated by thermal desorption analysis using a thermal-optical analyzer (Sunset Laboratory Inc.). PDMS standards were heated using a custom thermal desorption protocol using 12 temperature steps in He from 100 to 650 °C in 50 °C increments for 3 min each and one final step in O_2_ at 850 °C.

### 2.2 Aerosol Samples

Aerosol particles with a diameter <2.5 μm were sampled on filters in two tunnels in São Paulo, Brazil.^34,35^ These samples are referred to as aerosol samples in the remainder of the manuscript. First, 19 aerosol samples and corresponding field blanks were collected in the Jânio Quadros tunnel (Tunnel 1), which is located in the city center, 1.9 km long, with a speed limit of 60 km h^–1^. The sampling was conducted from May 4th to May 13th, 2011, and around 99% of the fleet were the light-duty vehicles during this period. Second, 13 aerosol samples and corresponding field blanks were collected in the RodoAnel Mário Covas tunnel (Tunnel 2), which is located in the city outskirts, 1.7 km long, with a speed limit of 70 km h^–1^. The sampling was conducted from July 6th to July 17th, 2011, and around 30% of the fleet were heavy-duty vehicles. Aerosol samples and field blanks were collected on quartz filters (precleaned at 800 °C for 12 h) at the midpoint of both tunnels. After sampling, they were wrapped in aluminum foil (precleaned at 550 °C for 8 h) and stored inside polyethylene bags in a freezer at −18 °C until analysis. This low-temperature preservation method is widely used in aerosol science to preserve reactive molecules in atmospheric aerosols. As an inert additive to lubricating oils used in high-temperature environments, the degradation of methylsiloxanes should be negligible at −18 °C. Tunnel parameters, fuel types, vehicle numbers, and sampling conditions can be found in Tables S8–S13.

### 2.3 Chemical Analysis

The chemical composition of the aerosol samples was analyzed using a thermal desorption-proton transfer reaction-time of the flight-mass spectrometer (TD-PTR-ToF-MS, PTR-TOF8000, Ionicon Analytik GmbH, Austria).^36,37^ The ionization method is proton transfer from H_3_O^+^ to organic molecules, and the ionized molecules are then detected by a time of flight-mass spectrometer with a mass resolution of 3000–4000 at full width at half-maximum. To prevent condensation of organic compounds, the drift tube and inlet line temperatures were controlled at 120 and 180 °C, respectively. The tunnel samples were thermally desorbed in an oven coupled to the PTR-ToF-MS using temperature steps of 3 min from 100 to 350 °C in 50 °C increments.^38^ Pure nitrogen was used as carrier gas at 100 mL min^–1^. Using thermal desorption, the total organic aerosols consist of desorbed and non-desorbed fractions. Organic aerosols (OA) in this study refer to desorbed organic aerosols, as we will not discuss the non-desorbed fraction.

The peak identification and integration were done by the PTRwid software,^39^ resulting in a concentration for each ion reported in ppb in the carrier gas. A unified mass list contains the mass-to-charge ratio (*m*/*z*) with ±2 sigma (95% confidence interval) for each ion, as well as possible molecular formulas that fall into this uncertainty range. The aerosol samples were measured three times, and the resulting values were subsequently reported as the arithmetic mean. The system blank or background was calculated by averaging eight mass spectra recorded, just before the heating of the sample in the oven started, i.e., with the sample in the oven and He flow through the system. The system blank was then subtracted from the raw data for each ion of each correlated sample including the field blank. Averages of field blanks (*n* ≥ 3) were subtracted from the sample mass spectra, and a 3σ limit of detection (LOD) was applied for each identified peak.

### 2.4 Identification and Quantification of Methylsiloxanes

Unlike the traditional gas chromatography coupled to mass spectrometry (GC-MS) methods, the identification and quantification of methylsiloxanes, in this study, were solely performed by mass spectrometry and the very characteristic stable isotope ratios of methylsiloxanes, as described in our previous study.^26^ Briefly, the identification of methylsiloxanes and derivatives relies on the isotope peaks derived from the heavier naturally occurring isotopes. For methylsiloxanes, these isotope peaks are more prominent than for organic compounds with CHON structures due to the high abundance of silicon isotopes: ^29^Si (*m*/*z* = 28.976 amu, 4.685%) and ^30^Si (*m*/*z* = 29.974 amu, 3.092%). For example, D_5_ (Decamethylcyclopentasiloxane, C_10_H_30_O_5_Si_5_) has the main peak at *m*/*z* = 371.102. The first isotope peak around *m*/*z* = 372.103 reaches 36.41% of the main peak height, and the second isotope peak around *m*/*z* = 373.104 reaches 23.56% of the main peak height. On the other hand, organics with CHON structures have smaller isotope peaks. For example, C_11_H_18_O_12_N_2_ has the molecular ion peak at *m*/*z* = 371.098 and cannot be distinguished from D_5_ at the mass resolution of the PTR-MS. However, for C_11_H_18_O_12_N_2_, the first isotope peak reaches only 13.29% of the main peak height and the second isotope peak only 3.25%. Therefore, the ratios of the first and second isotope peaks to the main peak were used for the identification of peaks of methylsiloxanes and derivatives and were further used in the quantification to eliminate interference from other compounds.^26^ D_1_–D_10_ methylsiloxanes in tunnel samples were statistically different from the field blank (Welch *t*-test, *p* = 0.0012), but D_11_–D_15_ methylsiloxanes were not (Welch t-test, *p* = 0.3070). Therefore, only D_1_–D_10_ methylsiloxanes were considered in the calculation of total concentration. Detailed information of identification and quantification in this study can be found in Supporting Information Section S1.

### 2.5 Emission Factors

The emission factors were calculated using eq 1.^34,35,40^
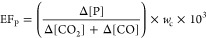
1EF_p_ refers to the emission factor of pollutant P in the unit of mg kg^–1^, i.e., mg of pollutant emitted per kg of fuel burned. Δ[P] refers to the enhancement of concentration of the pollutant (ng m^–3^) above the background. Δ[CO_2_] and Δ[CO] refer to the enhancement of the carbon concentrations of CO_2_ and CO in the unit of μg C m^–3^. *w*_c_ is the carbon mass fraction of the fuel (g C/g fuel).

Using eq 2, EF_p_ can be converted into EF_p_* in the unit of mg km^–1^ vehicle^–1^, meaning mg of pollutant emitted per km per vehicle.
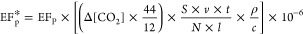
2*S* refers to the cross-sectional area of the tunnel (m^2^), *v* to the air velocity inside the tunnel (m s^–1^), *t* to the sampling time (s), *N* to the number of vehicles passing the tunnel during sampling, *l* to the tunnel length (m), ρ refers to the fuel density (g L^–1^), and *c* refers to the carbon intensity of the fuel (g CO_2_ L^–1^). The factor 44/12 refers to the unit conversion from g C to g CO_2_.

The vehicle type in Tunnel 1 was dominated by light-duty vehicles, which allows to estimate the emission factors of light-duty vehicles. Previous studies have shown that emissions from light-duty and heavy-duty vehicles have similar CO emission rates per kilometer.^34,35,41,42^ Therefore, Δ[CO_2_], Δ[CO], and Δ[P] of heavy-duty vehicles in Tunnel 2 can be estimated by subtracting emissions of light-duty vehicles (obtained from Tunnel 1) and used to calculate the emission factors of heavy-duty vehicles. The emission factors are referred to as EF_MS_ and EF*_MS_ for methylsilxoanes and EF_HC_ and EF*_HC_ for C_23_–C_38_ hydrocarbons from lubricating oils. The detailed equations and calculations can be found in Supporting Information Section S4.

## 3 Results

### 3.1 Methylsiloxanes in Particulate Vehicle Emissions

**Figure 1:**
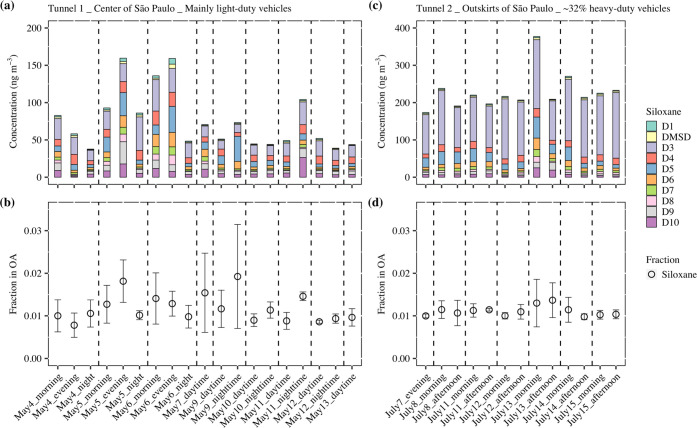
Concentrations of D_1_–D_10_ methylsiloxanes in the aerosol samples collected in (a) Tunnel 1 and (c) Tunnel 2. Mass fractions of methylsiloxanes in organic aerosols (OA) in (b) Tunnel 1 and (d) Tunnel 2.

Concentrations of D_1_–D_10_ methylsiloxanes from the tunnel samples are shown in Figure 1a,c (their fractions in total methylsiloxane mass are shown in Figure S2), and mass fractions of methylsiloxanes in organic aerosols (OA) are shown in Figure 1b,d. The identified methylsiloxanes were mainly small cyclic VMS (cVMS, (Si(CH_3_)_2_O)*_n_*; *n* = 3–15, D_3_–D_15_). Other fragments and derivatives include Si(CH_3_)_2_O (labeled as D_1_) and the monomer diol Si(CH_3_)_2_(OH)_2_ (dimethylsilanediol, DMSD). Oligomer diols (formed by hydrolysis, HO–(Si(CH_3_)_2_O)*_n_*–H) were not found in the tunnel samples but were identified in the ship emissions samples in our previous study.^26^ The absence of hydrolyzed products in tunnel samples suggested that the hydrolysis did not occur in TD-PTR-MS but can occur in marine engine combustion. Hydroxylated methylsiloxanes (wherein −CH_3_ is substituted by −OH) might be present in minor quantities, with their peaks overlapping the second isotope peaks of cVMS. In contrast, ship emissions exhibit an abundant presence of hydroxylated methylsiloxanes that were resolved from the second isotope peaks. These differences may be caused by the different engine construction and lubricating oils between vehicles and ships.

The concentrations of methylsiloxanes and derivatives in Tunnel 1 averaged 75.4 ± 39.2 ng m^–3^ (ranging from 37.7–160 ng m^–3^), corresponding to mass fractions in OA of 1.2 ± 0.3% (0.78–1.9%). The concentrations of methylsiloxanes and derivatives in Tunnel 2 averaged 229 ± 51 ng m^–3^ (174–377 ng m^–3^), corresponding to mass fractions in OA of 1.1 ± 0.1% (0.98–1.4%). Concentrations and mass fractions of individual methylsiloxanes can be found in Tables S3 and S4. The mass fractions of methylsiloxanes in OA were slightly higher in Tunnel 1 (mainly light-duty vehicles) than in Tunnel 2 (around 32% heavy-duty vehicles). The consistent mass fraction of methylsiloxanes within OA observed in both tunnels indicated a close temporal correlation between the fluctuations of methylsiloxanes and OA. The mass fractions of methylsiloxanes in OA within the tunnels were comparable to the approximately 1.2% detected in ship emissions during stable operation in our previous study.^26^ However, during transient engine states of the ship, such as acceleration, deceleration, and standby, the content of methylsiloxanes in OA significantly increased in ship emissions, ranging from 28.2 to 59.3%. This may be related to the different lubrication of the marine engine, resulting in more methylsiloxane emissions during inefficient combustion or idling conditions. In general, more lubricating oil enters the combustion cylinder and stays there longer in marine engines than in vehicle engines, details in Supporting Information Section S5.

### 3.2 Molecular Size of Methylsiloxanes in Particulate Vehicle Emissions

**Figure 2:**
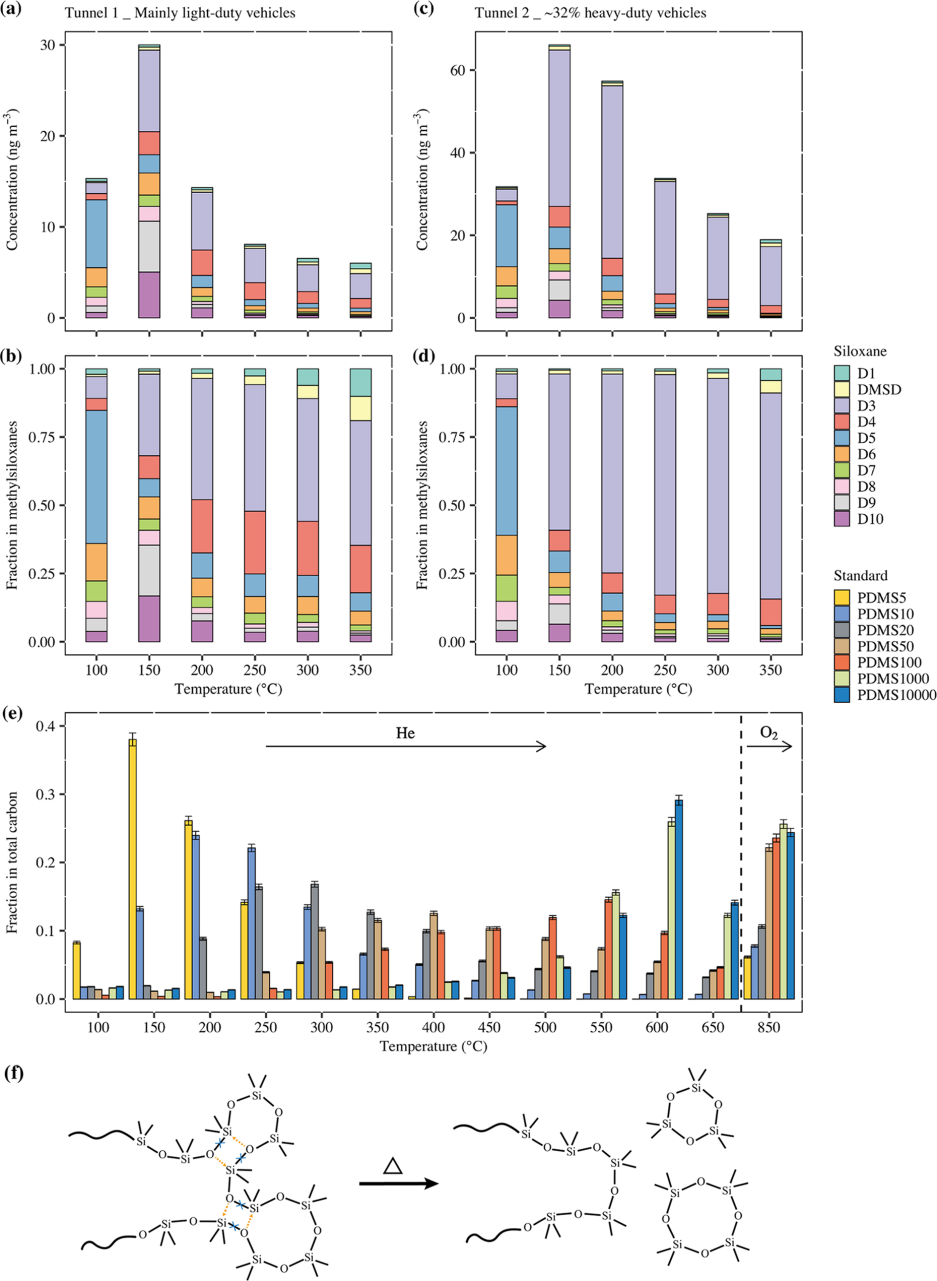
Temperature dependence of methylsiloxane desorption from filter samples. (a, c) Average concentrations of methylsiloxanes desorbed at elevated temperature steps from aerosol filter samples collected in Tunnel 1 and Tunnel 2. (b) and (d) Mass fractions of D_1_–D_10_ in total detected methylsiloxanes for Tunnel 1 and Tunnel 2. Detailed concentrations and fractions are shown in Figures S5–S8 and Table S7. (e) Thermal desorption of commercially available PDMS standards at elevated temperature steps first in He and then in O_2_, in a thermal-optical analyzer. The methylsiloxanes are quantified by their carbon mass and shown at each step as a fraction of total carbon in the sample. See data in Table S6. (f) Schematic of PDMS depolymerization under heating.

Figure 2a,c shows the average concentrations of desorbed methylsiloxanes and derivatives detected in each tunnel at elevated desorption temperatures, and Figure 2b,d shows the fractional contribution of various compounds to total methylsiloxane mass at each temperature step. In both tunnels, the composition of methylsiloxanes at the 100 °C step was dominated by D_5_ (around 50%, volatile), which was different from the other temperature steps. The fractions of D_8_–D_10_ (less volatile) in the detected methylsiloxanes were highest in the 150 °C step for both tunnels. These methylsiloxane molecules may have been emitted directly in the particle phase or condense onto them after cooling down of the vehicle emissions. In the 200–350 °C steps, D_1_–D_4_ accounted for 67–94% of the total methylsiloxanes in the tunnels. It is not likely that these small molecules would desorb at higher temperatures than their larger counterparts (D_5_–D_10_). Moreover, due to their higher vapor pressures, DMSD and D_3_–D_4_ usually do not partition to the particle phase. Therefore, the small molecules detected in the 200–350 °C steps were more likely fragments from large molecules of methylsiloxanes, e.g., PDMS contained in the particles.

Previous studies show that PDMS can undergo depolymerization at high temperatures and fragment into smaller cVMS.^26,43−47^ The product yields are usually in decreasing concentrations from D_3_ to D_4_, D_5_, D_6_, and larger methylsiloxanes. The schematic of PDMS depolymerization under heating is shown in Figure 2f. Siloxane bond rearrangement leads to the expulsion of the cVMS molecules and shortening of the remaining chain, which may be related to the participation of silicon d orbitals.^26,43−47^ Transition states can be formed at any point in the chain, and the process can proceed indefinitely within the chain until the remaining linear structure is too short to form a cyclic molecule, explaining the descending order of product concentration versus molecular size.

Based on the thermal desorption and depolymerization properties of PDMS, a new method was designed to estimate the molecular size of methylsiloxanes in aerosol samples by comparing their thermal desorption properties with PDMS standards of different molecular sizes. Commercially available PDMS samples with different viscosities were thermally desorbed at elevated temperatures, and the desorbed carbon mass is shown as a fraction of total carbon in Figure 2e. The PDMS5, PDMS10, PDMS20, PDMS50, PDMS100, PDMS1000, and PDMS10000 correspond to approximate molecular sizes of 8, 15, 25, 50, 80, 400, and 800 siloxane units, respectively.^33^ In general, oligomer methylsiloxanes (e.g., PDMS5, PDMS10) mainly desorbed or decomposed at temperatures lower than 300 °C, while polymer methylsiloxanes (e.g., PDMS1000, PDMS10000) mainly desorbed or decomposed at temperatures higher than 300 °C. Some previous studies have also demonstrated the high thermal stability of polymer methylsiloxanes.^48−50^

Our hypothesis is that polymer methylsiloxanes derived from fuel oils or lubricating oils can break down into various molecular sizes in engine combustion. According to the comparison of the thermal desorption and depolymerization patterns of Figure 2a–e, the average molecular size of methylsiloxanes in tunnel samples might be between 8–15 siloxanes units (relatively larger in Tunnel 2 for heavy-duty vehicles). However, the presence of larger methylsiloxanes that desorb at temperatures >350 °C is also possible, including the original PDMS. Moreover, depolymerization properties can be used to distinguish the initial form of methylsiloxane molecules in tunnel samples. In Figure 2a–d, the dominance of D_5_–D_10_ in the 100 °C step implies that methylsiloxanes in this step were mainly desorbed rather than depolymerized, otherwise D_3_–D_4_ should be higher. On the other hand, predominant D_3_–D_4_ in the 150 °C step indicates that depolymerization was starting to play a leading role, but the considerable D_8_–D_10_ fractions mean that thermal desorption still played a role in this step. In the 200–350 °C steps, considerable D_3_–D_4_ fractions indicate that depolymerization dominated. Overall, the methylsiloxanes in the low-temperature steps mainly originated from thermal desorption, and the methylsiloxanes in the high-temperature steps mainly originated from depolymerization. In addition, the proportions of D_1_ and DMSD in methylsiloxanes increased with temperature, indicating that large molecular methylsiloxanes tended to generate more small monomer fragments. Upon reviewing our previous research results, we discovered that ship emissions also exhibit a similar trend,^26^ indicating that the cVMS detected at high temperatures should also be attributed to the depolymerization of long-chain methylsiloxanes.

### 3.3 Methylsiloxanes and Lubricating Oils

**Figure 3:**
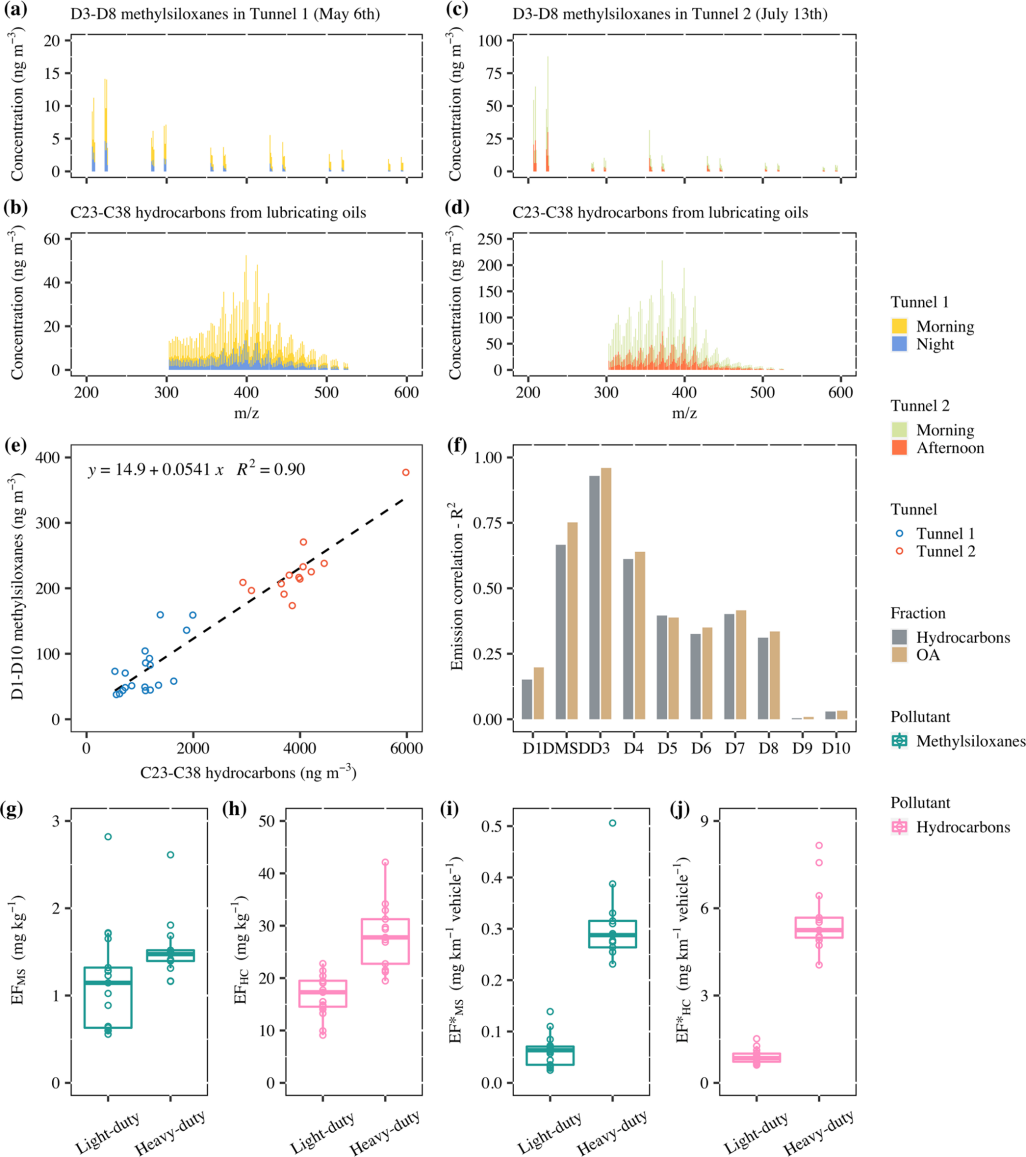
(a–d) Mass spectra of D_3_–D_8_ methylsiloxanes and C_23_–C_38_ hydrocarbons from lubricating oils in aerosol samples collected in Tunnel 1 and Tunnel 2. (e–f) Relationships between D_1_–D_10_ methylsiloxanes, C_23_–C_38_ hydrocarbons, and organic aerosols (OA). The black dashed line is the linear fit. (g–j) Emission factors of D_1_–D_10_ methylsiloxanes and C_23_–C_38_ hydrocarbons of light-duty and heavy-duty vehicles.

In addition to methylsiloxanes, typical C_23_–C_38_ hydrocarbons from lubricating oils were identified in the mass spectra (which differ from C_5_–C_12_ hydrocarbons typical for gasoline and from C_12_–C_20_ typical for diesel fuels), as shown in Figure 3a–d. This indicates that unburned lubricating oils contributed to particulate emissions from vehicle engine combustion. Lubricating oils in turn might contain methylsiloxanes as antifoam additives. The mass spectra of D_3_–D_8_ methylsiloxanes and C_23_–C_38_ hydrocarbons exhibited notable differences between the two tunnels. Methylsiloxanes in Tunnel 2 contained a larger fraction of D_3_, and C_23_–C_38_ hydrocarbons in Tunnel 1 had on average higher *m*/*z* ratios than in Tunnel 2. These discrepancies could indicate variations in the lubricating oils used for light-duty and heavy-duty engines. While absolute concentrations varied with the time of the day, these distinctive features of the mass spectra remained the same in each tunnel. On average, the concentrations of C_23_–C_38_ hydrocarbons were 1100 ± 420 ng m^–3^ in Tunnel 1 and 3980 ± 730 ng m^–3^ in Tunnel 2, and the corresponding mass fractions in OA were 18 ± 4% in Tunnel 1 and 19 ± 2% in Tunnel 2. See data for all samples in Tables S3 and S4.

Figure 3e shows that the concentration of D_1_–D_10_ methylsiloxanes was correlated with the concentrations of C_23_–C_38_ hydrocarbons (*R*^2^ = 0.90, *p*-value = 2.2 × 10^–16^), and the concentration of D_1_–D_10_ methylsiloxanes was also closely related to OA (*R*^2^ = 0.94, *p*-value = 2.2 × 10^–16^, Figure S9a), implying their main source is from vehicle emissions (fuel oils or lubricating oils) rather than other sources (such as coating, tire wear, and personal care products by the passengers). The correlation between C_23_–C_38_ hydrocarbons and OA was even stronger (*R*^2^ = 0.97, p-value <2.2 × 10^–16^, Figure S9b), so C_23_–C_38_ hydrocarbons in lubricating oils (around 18% of desorbed OA) may be a significant part of vehicle emissions to the atmosphere. In addition, the slope of the fit can be recognized as an approximated ratio, and the positive intercept in Figure 3e (and Figure S9a) indicated other potential sources of methylsiloxanes, although in small amounts. In the detailed correlation analysis depicted in Figure 3f, DMSD, D_3_, and D_4_ exhibited stronger associations with C_23_–C_38_ hydrocarbons and OA compared to other methylsiloxanes. Notably, D_3_ demonstrated a particularly high correlation with C_23_–C_38_ hydrocarbons (*R*^2^ = 0.93, *p*-value <2.2 × 10^–16^) and OA (*R*^2^ = 0.96, *p*-value <2.2 × 10^–16^). Since we showed above that D_3_ was a main depolymerization fragment of polymer methylsiloxanes produced during TD-PTR-MS analysis, the high correlations with C_23_–C_38_ hydrocarbons and OA suggest that the large methylsiloxane molecules in the particulate matter were from vehicle emissions, and the semi-volatile methylsiloxanes desorbed at the low-temperature steps might have other sources in addition.

Numerous studies have focused on investigating specific compounds present in vehicle emissions, such as polycyclic aromatic hydrocarbons (PAHs), N-containing compounds, and nitrated phenols,^51−53^ and integrating this knowledge can potentially shed light on the emissions of methylsiloxanes from engines. While the molecular composition of each compound can be identified using the resolution of PTR-MS, determining the exact molecular structure remains challenging. We categorized the desorbed organics based on their elemental composition (CH, CHO, CHON, and CHN compounds) and examined their correlation with methylsiloxanes. The resulting *R*^2^ values ranged from 0.91 to 0.93 (Figure S10), indicating that there is no significant association between methylsiloxanes and any specific compound class.

The high correlation with OA provides a rationale for reporting emission factors relative to the consumption of fuel oils. The emission factors of D_1_–D_10_ methylsiloxanes and C_23_–C_38_ hydrocarbons are shown in Figure 3g–j. See detailed results in Tables S11 and S13. The emission factors of D_1_–D_10_ methylsiloxanes were on average 1.16 ± 0.59 mg kg^–1^ (or 0.0619 ± 0.0300 mg km^–1^ vehicle^–1^) for light-duty vehicles and 1.53 ± 0.37 mg kg^–1^ (or 0.306 ± 0.072 mg km^–1^ vehicle^–1^) for heavy-duty vehicles. The emission factors of methylsiloxanes per unit fuel consumption of light-duty and heavy-duty vehicles were very similar, which supports the possibility that the methylsiloxanes were derived from the fuel oils. However, whether the methylsiloxanes come from fuel oils, lubricating oils, or both, still needs further research. The emission factors of methysiloxanes per unit kilometer per heavy-duty vehicle was significantly higher than that of light-duty vehicles, which might be related to the consumption differences of fuel oils or lubricating oils. The emission factors of C_23_–C_38_ hydrocarbons were on average 16.7 ± 3.9 mg kg^–1^ (or 0.895 ± 0.240 mg km^–1^ vehicle^–1^) for light-duty vehicles and 28.2 ± 6.2 mg kg^–1^ (or 5.61 ± 1.15 mg km^–1^ vehicle^–1^) for heavy-duty vehicles. This is comparable to emission factors of PAHs with *m*/*z* <300, corresponding to hydrocarbons <C_22_ reported by previous studies for vehicles (e.g., 0.073–0.090 mg kg^–1^ of PAHs for light-duty vehicles, 0.014–2.3 mg kg^–1^ for heavy-duty vehicles).^40,54,55^ In this study, C_23_–C_38_ hydrocarbons accounted for around 18% of OA on average, but hydrocarbons <C_22_ (including unburned fuel oils, PAHs, and other compounds) only accounted for around 14% of OA. Previous literature also reported the presence of lubricating oils in traffic emissions,^56,57^ but it did not attract much attention.

## 4 Discussion

Methylsiloxanes were found in tunnel aerosol samples in this study, accompanied by significant amounts of C_23_–C_38_ hydrocarbons from lubricating oils. The mass fractions of methylsiloxanes in desorbed OA were estimated on average 1.2% (±0.3%) and 1.1% (±0.1%) for the two tunnels, respectively. In a previous tunnel study, thermally desorbed organic carbon up to 350 °C accounted for 40.2 ± 3.2% of total organic carbon.^58^ We assume that roughly the same fraction holds for desorbed OA versus total OA for the tunnel samples in this study. Total organic aerosols account for 26–29^59^ and 39–42%^60^ of the aerosol in other tunnel studies. Based on the aforementioned assumptions, the estimated mass fraction of methylsiloxanes in aerosols (i.e., the whole particles comprising organic and inorganic matter) falls within the range of 0.1 to 0.2%. As a widespread source, vehicle emissions may contribute significantly to particulate methylsiloxanes in the ambient atmosphere, especially in urban environments.

The oxidation of gas-phase methylsiloxanes and the related secondary aerosol formation has received increasing attention in recent years. Particle size and morphology,^23^ OH concentration,^61^ and chlorine chemistry^62^ were found to influence the oxidation of methylsiloxanes. The global annual production of D_4_, D_5_, and D_6_ is estimated to be 1, 0.1, and 0.01 Tg yr^–1^, and most of these cVMS are eventually released into the atmosphere.^63−67^ The main oxidation products of cVMS are siloxanols,^68^ which remain mainly in the gas phase. Only a small fraction of cVMS oxidation products enter the particle phase, as recent modeling suggests that the annual SOA production of D_4_–D_6_ is around 0.01 Tg yr^–1^.^69^

The emission of methylsiloxanes from vehicles may vary by region and environment, which is further related to the number, type, and age of vehicles, as well as the production and consumption of fuel oils and lubricating oils. Emission factors in the tunnels should be representative of a certain degree of mixture of different types and ages of vehicles, fuel oils, and lubricating oils, so they can serve a rough estimate.

According to the Oil Market Report in 2022 by International Energy Agency (IEA),^70^ the global demand of oil averaged 97.6 million barrels per day (approximately 4.84 × 10^3^ Tg yr^–1^) during 2020–2023, and motor gasoline and gas/diesel oil account for on average 25.9% (1.26 × 10^3^ Tg yr^–1^) and 28.2% (1.36 × 10^3^ Tg yr^–1^), respectively. Using the EF in the unit of mg kg^–1^, the global annual emissions of methylsiloxanes are estimated at 0.0015 ± 0.0007 and 0.0021 ± 0.0005 Tg yr^–1^ for light-duty and heavy-duty vehicles. The global annual emissions of hydrocarbons from lubricating oils are estimated at 0.021 ± 0.005 and 0.038 ± 0.009 Tg yr^–1^ for light-duty and heavy-duty vehicles. This is comparable to the estimate of secondary production of particle-phase methysiloxanes cited above.

Based on the data from the U.S. Department of Energy,^71^ the number of vehicles worldwide are 1.08 × 10^9^ and 4.07× 10^8^ for light-duty and heavy-duty vehicles in 2019. According to the vehicle miles traveled reported by HIS Markit in the main oil consumption country worldwide,^72^ the annual vehicle traveled distance is 2.07 × 10^4^ and 1.87 × 10^4^ km for light-duty and heavy-duty vehicles on average, which can be recognized as worldwide estimates. These figures are close to the U.S. Department of Energy’s 2019 national figures (only in U.S.) of 2.04 × 10^4^ and 1.64 × 10^4^ km for light-duty and heavy-duty vehicles.^71^ Using the EF* in the unit of mg km^–1^ vehicle^–1^, the annual emissions of methylsiloxanes are estimated at 0.0022 ± 0.0011 and 0.0038 ± 0.0009 Tg yr^–1^ for light-duty and heavy-duty vehicles, respectively. The global annual emissions of hydrocarbons from lubricating oils are estimated at 0.032 ± 0.009 and 0.069 ± 0.014 Tg yr^–1^ for light-duty and heavy-duty vehicles. While estimates of annual emissions based on vehicle number and traveled distance are slightly higher than those based on fuel consumption, both estimation methods are within the same order of magnitude. Combining both methods and vehicle types, vehicles emit approximately 0.0035–0.0060 Tg yr^–1^ of methylsiloxanes and 0.059–0.10 Tg yr^–1^ of hydrocarbons from lubricating oils into the atmosphere, with a significant portion being released in urban areas. The annual methylsiloxane emissions in the particle phase by engines should be higher than this estimate because there are other industrial and marine engines.

Given the particle-phase concentrations of methylsiloxanes observed in this study, it is likely that vehicles also emit methylsiloxanes in the gas phase, especially if PDMS fragments during engine combustion. The fragmentation may be affected by vehicle type, combustion condition, as well as engine wear and efficiency. Future studies focused on the gas-particle phase partitioning of vehicle-emitted methylsiloxanes may shed further light on this.

We provided conclusive evidence of methylsiloxanes in aerosol particles from vehicle emissions, likely related to fuel oils or lubricating oils. Lubricating oils and their additives, including antifoams, are inevitable for normal engine operation. Methylsiloxanes are poisonous to human beings or harmful to the environment, but other antifoams can be worse, such as fluorine-containing compounds.^73^ More studies are needed to evaluate the health effects of methylsiloxanes before their adverse effects relative to other antifoams can be assessed. In addition, methylsiloxanes are good surfactants, so their role in atmospheric physical and chemical reactions needs to be determined.

## 5 Atmospheric Implications

Due to the global distribution of vehicle emissions, humans may have been exposed to methylsiloxanes for long periods of time. A wide variety of methylsiloxanes could be emitted by vehicles, including VMS, hydroxylated methylsiloxanes, hydrolyzed methysiloxanes, and methylsiloxanes with molecular sizes between VMS and PDMS. Byproducts of methylsiloxane combustion, such as nanosilica, can be another potential contamination that residents inhale. The effects on human health, especially the long-term effects, require urgent research.

The role of methylsiloxanes in atmospheric aerosols is virtually unknown. Since methylsiloxanes are good surfactants, their role in atmospheric physical and chemical reactions, such as cloud condensation nuclei and ice nuclei, needs to be determined. The impact of 0.1–0.2% methylsiloxanes in aerosols should not be overlooked, as they have the potential to reduce surface tension and potentially lead to a decrease in the particle size required for cloud droplet activation. As a relatively stable material, methylsiloxanes are not easily oxidized, but existing studies have shown that VMS still participate in the atmospheric oxidation process. Therefore, the particle-phase oxidation of methylsiloxanes also needs to be investigated.

Comparing to the gas-to-particle conversion source, the annual methylsiloxane emissions from vehicle-emitted aerosols are considerable and cannot be ignored. However, the difference between residential emissions and traffic emissions of methylsiloxanes is still unclear. It currently remains challenging to determine the source and proportion of atmospheric methylsiloxanes, so we are still unable to conduct source apportionment for methylsiloxanes. Due to the stable physical and chemical properties of methylsiloxanes, long lifetimes in the atmosphere can be expected but still need to be determined. Methylsiloxanes are man-made chemicals with no natural source, and therefore, methylsiloxanes are indicators of anthropogenic sources, especially in remote areas, such as, glaciers, oceans, and polar regions.

## Supporting Information Available

Identification and quantification of methylsiloxanes; molecular sizes of methylsiloxanes in tunnel aerosols compared to commercial PDMS; correlation between organic compounds and methylsiloxanes in traffic emissions; emission factors calculations and intermediate results; differences in vehicle and marine engine lubrication (PDF)
