# Supplementary material for: Methylsiloxanes from Vehicle Emissions Detected in Aerosol Particles
Source: Environ Sci Technol. 2023 Sep 12;57(38):14269–79. doi: 10.1021/acs.est.3c03797 (PMC10537456; doi:10.1021/acs.est.3c03797)
Supplement: Supplementary file 1 — es3c03797_si_001.pdf [file es3c03797_si_001.pdf]

Supporting Information

Methylsiloxanes from vehicle emissions detected in aerosol particles

Peng Yao [1], Rupert Holzinger [2], Dušan Materić [2,5], Beatriz Sayuri Oyama [2,3], Maria de Fátima Andrade [3], Dipayan Paul [1], Haiyan Ni [1,4], Hanne Noto [2], Ru-Jin Huang [4], Ulrike Dusek [1,*]

[1] Centre for Isotope Research (CIO), Energy and Sustainability Research Institute Groningen (ESRIG), University of Groningen, Groningen, 9747 AG, the Netherlands

[2] Institute for Marine and Atmospheric Research, IMAU, Utrecht University, Princetonplein 5, 3584 CC, Utrecht, the Netherlands

[3] Institute of Astronomy, Geophysics and Atmospheric Sciences, University of São Paulo, São Paulo 05508-090, Brazil

[4] State Key Laboratory of Loess and Quaternary Geology, Center for Excellence in Quaternary Science and Global Change, Key Laboratory of Aerosol Chemistry & Physics, Institute of Earth Environment, Chinese Academy of Sciences, Xi'an 710061, China

[5] Department of Analytical Chemistry, Helmholtz Centre for Environmental Research – UFZ, Permoserstrasse 15, 04318 Leipzig, Germany

Corresponding author: u.dusek@rug.nl (Ulrike Dusek)

Number of Pages: 36

Number of figures: 10

Number of tables: 13

S1. Identification and quantification of methylsiloxanes

The identification and quantification of methylsiloxanes and derivatives were conducted based on a method described in our previous study.[1] The methylsiloxane peaks identified in the tunnel samples are shown in Table S1a, including the main peak (also called base peak), main+1, and main+2 isotope peaks. Most of the peaks were consistent with the theoretical m/z value, validating the presence of various methylsiloxanes. Some peaks deviated slightly from the theoretical m/z value, which might be due to peak identification and integration bias, interference of other compounds with similar m/z values, and instrument calibration.

The theoretical (M+1)/M and (M+2)/M ratios between the main peak and isotope peaks are shown in Table S2a. The identified methylsiloxanes were mainly small cVMS (D_3–D_{15}) and their positively charged fragments (D_3f–$D_{15}f$), produced during ionization by loss of one –CH_3. These positively charged fragments were added to the mass of the corresponding cVMS for easy understanding. As described in our previous study, (M+1)/M ratios of CHON compounds with similar m/z values are much lower than methylsiloxanes.[1] For example, D_5 has m/z = 371.102 and (M+1)/M = 0.3641, and other possible compounds are $C_{11}H_{18}O_{12}N_2H^+$ (371.094), $C_{16}H_{18}O_{10}H^+$ (371.098), $C_{15}H_{18}O_9N_2H^+$ (371.109), and $C_{13}H_{22}O_{12}H^+$ (371.119) with (M+1)/M ratios ranging from 0.1329 to 0.1789. Based on the uncertainty of the PTR-MS quantification, we consider (M+1)/M ratios that fall within the range of 70% to 130% of the theoretical ratio as indicative methylsiloxanes, since this range does not overlap with that of possible CHON compounds. For verification, the (M+1)/M ratios of D_3–D_{15} and D_3f–$D_{15}f$ were checked in the raw data before blank subtraction, and on average 49% and 48% of the (M+1)/M ratios fell within the valid range for Tunnel 1 and Tunnel 2, respectively. Since about half of the large number of ratios met the requirements (26 × 33 × 3 × 6 ratios in total; 26 methylsiloxanes; 20 samples in Tunnel 1 and 13 samples in Tunnel 2; 3 replicates; 6 temperature steps), the presence of methylsiloxanes was confirmed. For D_1 and DMSD with only one Si atom in the molecule, the (M+1)/M ratios are not significantly higher than for other CHON structures, so the verification method above does not work effectively. We identified the

existence of D_1 and DMSD for three reasons: first, the detected m/z values were in good agreement with the theoretical values; second, they are reasonable fragments and products of methylsiloxanes formed in PTR-MS; third, substantial amounts of D_1 and DMSD were also found in our previous study on ship emissions.[1]

The detected median (M+1)/M and (M+2)/M ratios of D_1–D_{15} are shown in Table S2a. The median (M+1)/M ratios of D_3–D_{15} were mostly close the theoretical ratios, but (M+2)/M ratios were sometimes higher than the theoretical ratios. Higher (M+2)/M ratios might be caused by the oxidation products of methylsiloxanes in the PTR-MS, for example, the main+2 peak of D_3 has a m/z value of 225.061–225.071, and associated oxidation product D_2T^{OH} ($C_5H_{16}O_4Si$) has a m/z value of 225.043. These two peaks are sometimes not separated by PTR-MS resolution, which ultimately contribute to a higher detected main+2 peak. In addition, the (M+1)/M and (M+2)/M ratios of minor peaks in some samples (close to the detection limit) can be influenced by background, peak integration, and other compounds with close m/z values. To minimize these interferences, concentration corrections were conducted for each set of methylsiloxane peaks. The main idea of the concentration correction is to include the main and isotope peaks without interferences. The main peak, main+1 isotope peak, and (M+1)/M ratio were used for correction in this study. Specifically, if the (M+1)/M ratio is higher than the theoretical ratio, the main peak is considered less interfered, and interfering compounds are more likely to be present on the main+1 isotope peak. Otherwise, the main+1 isotope peak is considered less interfered. Then the concentration can be calculated by dividing the peak with less interference by the theoretical fraction of this particular peak in the total amount of the substance (including the main peak and all isotope peaks). The theoretical fractions of the main peaks and isotope peaks in the total amount of the substance are also shown in Table S2a. The quantification of methylsiloxanes in mixtures is inherently difficult, the interference can only be minimized, not eliminated. For an easier understanding, the fragments D_3f–D_{15}f were combined with relevant main compounds (D_3–D_{15}) in the calculation and presentation of the results.

Hydroxylated methylsiloxanes, where a methyl group ($–CH_3$) is replaced by a hydroxyl group ($–OH$), have been identified in ship emissions.[1] However, in the mass spectra of vehicle samples, the related peaks were either not present or maybe insufficiently resolved from the isotope peaks of cVMS in Table S1a–S2a. For instance, the detected peak at m/z = 225.053 was likely a composite signal resulting from the overlap of the main+2 peak of D_3 (m/z = 225.061–225.071) and the main peak of D_2T^{OH} (m/z = 225.043). The hydroxylated peaks were likely not fully resolved due to relatively low concentrations, but can tentatively be hypothesized due to the shift of the observed main+2 peak of e.g. D_3 to slightly smaller masses (see Table S1b) than expected based on theory. In addition, the (M+2)/M ratios (see Table S2a) were higher than theoretically predicted, indicating an additional compound was present at the M+2 mass. This indirect evidence was consistent with the presence of lower molecular weight hydroxylated methylsiloxanes (D_2T^{OH}–D_5T^{OH}) and corresponding fragments ($D_2T^{OH}f$–$D_5T^{OH}f$) as shown in Table S1b–S2b. For cVMS of higher molecular weight, the (M+2)/M values in Table 2a, were much closer to their theoretical (M+2)/M ratios. Thus, hydroxylated compounds were therefore likely not present at methylsiloxanes larger than D_5 or in relatively small quantities. PDMS standard samples analyzed by PTR-MS exhibited hydroxylated fragments as well,[1] so the origin of these hydroxylated methylsiloxanes cannot be unequivocally attributed to oxidation in the vehicle engine or the TD-PTR-MS ionization process.

The hydroxylated methylsiloxanes were not well resolved and only accounted for a small proportion in the vehicle samples, but excluding these compounds may underestimate the vehicle emissions. Therefore, these hydroxylated methylsiloxanes were included in the overall emission calculation, analogous to D_1 and DMSD. Considering the peak overlapping, the determination of concentration becomes more difficult. The detailed procedure involved initially computing the concentrations of main+2 and main+3 of D_3–D_6 and D_3f–D_6f methylsiloxanes according to their theoretical ratios, as presented in Table 1a–2a. Then these calculated concentrations were subtracted from the measured peaks at the relevant masses, yielding the concentrations of the main and main+1 peaks of D_2T^{OH}–D_5T^{OH} and $D_2T^{OH}f$–$D_5T^{OH}f$.

Thereafter, the (M+1)/M ratio of D_2T^{OH}–D_5T^{OH} and $D_2T^{OH}f$–$D_5T^{OH}f$ can be utilized to estimate their concentrations as described above. The adoption of a conservative calculation approach guarantees that the calculation is skewed towards underestimation and can effectively mitigate the potential bias emanating from these not well-resolved hydroxylated methylsiloxanes.

Table S1a. The theoretical and detected m/z values of the main peak and main+1, main+2 isotope peaks of methylsiloxanes in aerosol samples.

Siloxane	Theoretical m/z values			Detected m/z values		
	Main	Main+1	Main+2	Main	Main+1	Main+2
D_1	75.027	76.026–76.031	77.023–77.033	75.028	76.029	77.022
DMSD	93.037	94.037–94.041	95.034–95.044	93.036	94.033	na
D_3f	207.033	208.032–208.037	209.030–209.040	207.034	208.035	209.022
D_3	223.064	224.064–224.068	225.061–225.071	223.065	224.067	225.053
D_4f	281.052	282.051–282.056	283.049–283.058	281.053	282.054	282.049
D_4	297.083	298.083–298.087	299.080–299.090	297.083	298.084	299.079
D_5f	355.070	356.070–356.075	357.067–357.077	355.071	356.072	357.068
D_5	371.102	372.101–372.106	373.099–373.108	371.103	372.103	373.099
D_6f	429.089	430.089–430.093	431.086–430.096	429.090	430.090	431.085
D_6	445.121	446.120–446.125	447.117–447.127	445.121	446.120	447.116
D_7f	503.108	504.108–504.112	505.105–505.115	503.107	504.106	505.100
D_7	519.139	520.139–520.144	521.136–521.146	519.137	520.137	521.130
D_8f	577.127	578.126–578.131	579.124–579.134	577.124	578.126	579.115
D_8	593.158	594.158–594.162	595.155–595.165	593.154	594.154	595.144
D_9f	651.146	652.145–652.150	653.142–653.152	651.138	652.141	653.134
D_9	667.177	668.177–668.181	669.174–669.184	667.171	668.169	669.164
D_{10}f	725.164	726.164–726.169	727.161–727.171	725.156	726.154	727.151
D_{10}	741.196	742.195–742.200	743.193–743.202	741.186	742.183	743.184
D_{11}f	799.183	800.183–800.187	801.180–801.190	799.171	800.169	801.162
D_{11}	815.215	816.214–816.219	817.211–817.221	815.201	816.200	817.197
D_{12}f	873.202	874.202–874.206	875.199–875.209	873.184	874.184	875.179
D_{12}	889.233	890.223–890.238	891.230–891.240	889.222	890.217	891.214
D_{13}f	947.221	948.220–948.225	949.218–949.228	947.199	948.197	949.196
D_{13}	963.252	964.252–964.256	965.249–965.259	963.227	964.228	965.226
D_{14}f	1021.240	1022.239–1022.244	1023.236–1023.246	1021.213	1022.219	1023.215
D_{14}	1037.271	1038.270–1038.275	1039.268–1039.278	1037.244	1038.247	1039.249
D_{15}f	1095.258	1096.258–1096.263	1097.255–1097.265	1095.225	1096.225	1097.228
D_{15}	1111.290	1112.289–1112.294	1113.287–1113.296	1111.257	1112.251	1113.255

Table S1b. The theoretical m/z values of the main peak and main+1, main+2 isotope peaks of hydroxylated methylsiloxanes in aerosol samples.

Siloxane	Theoretical m/z values		
	Main	Main+1	Main+2
$D_2T^{OH}f$	209.012	210.012–210.016	211.009–211.019
D_2T^{OH}	225.043	226.043–226.048	227.040–227.050
$D_3T^{OH}f$	283.031	284.031–284.035	285.028–285.038
D_3T^{OH}	299.062	300.062–300.066	301.059–301.069
$D_4T^{OH}f$	357.050	358.049–358.054	359.047–359.056
D_4T^{OH}	373.081	374.081–374.085	375.078–375.088
$D_5T^{OH}f$	431.069	432.068–432.073	433.065–433.075
D_5T^{OH}	447.100	448.099–448.104	449.097–449.107

Table S2a. The theoretical fractions of main peaks and isotope peaks in the total amount of the substance, the theoretical (M+1)/M and (M+2)/M ratios between main peak and isotope peaks, and the detected median (M+1)/M and (M+2)/M ratios in two tunnels of methylsiloxanes in aerosol samples (D_1–D_3 in the 150 °C fraction and D_4f–D_{15} in the 100 °C fraction).

Siloxane	Fractions				Theoretical ratios		Detected median ratios	
	Main	Main+1	Main+2	Rest	(M+1)/M	(M+2)/M	(M+1)/M	(M+2)/M
D_1	0.9004	0.0656	0.0331	0.0009	0.0728	0.0368	0.1039	0.3437
DMSD	0.8982	0.0657	0.0349	0.0011	0.0732	0.0389	0.5209	1.2907
D_3f	0.7379	0.1532	0.0914	0.0175	0.2076	0.1239	0.2475	0.4016
D_3	0.7300	0.1595	0.0921	0.0185	0.2184	0.1261	0.4816	0.7792
D_4f	0.6644	0.1863	0.1167	0.0326	0.2804	0.1757	0.3297	0.3849
D_4	0.6573	0.1914	0.1174	0.0338	0.2913	0.1787	0.3567	0.5404
D_5f	0.5982	0.2113	0.1392	0.0513	0.3533	0.2327	0.3535	0.2884
D_5	0.5918	0.2155	0.1399	0.0528	0.3641	0.2365	0.3518	0.3260
D_6f	0.5386	0.2295	0.1588	0.0730	0.4261	0.2949	0.4083	0.3286
D_6	0.5329	0.2328	0.1596	0.0747	0.4369	0.2995	0.4482	0.3533
D_7f	0.4850	0.2420	0.1758	0.0973	0.4989	0.3624	0.5385	0.5623
D_7	0.4798	0.2446	0.1765	0.0992	0.5097	0.3678	0.5340	0.5171
D_8f	0.4367	0.2497	0.1900	0.1236	0.5717	0.4351	0.6398	0.6650
D_8	0.4320	0.2517	0.1906	0.1257	0.5825	0.4413	0.5936	0.6355
D_9f	0.3932	0.2534	0.2018	0.1516	0.6445	0.5131	0.6766	0.6558
D_9	0.3890	0.2549	0.2023	0.1538	0.6553	0.5201	0.6576	0.6623
D_{10}f	0.3540	0.2540	0.2111	0.1809	0.7173	0.5964	0.7551	0.7041
D_{10}	0.3503	0.2550	0.2116	0.1831	0.7281	0.6041	0.7320	0.6918
D_{11}f	0.3188	0.2519	0.2183	0.2110	0.7901	0.6848	0.8100	0.7360
D_{11}	0.3154	0.2526	0.2187	0.2134	0.8009	0.6933	0.7880	0.7337
D_{12}f	0.2870	0.2477	0.2235	0.2418	0.8629	0.7786	0.8675	0.8073
D_{12}	0.2840	0.2481	0.2237	0.2442	0.8738	0.7878	0.8456	0.7850
D_{13}f	0.2584	0.2418	0.2268	0.2729	0.9358	0.8775	0.9393	0.8907
D_{13}	0.2557	0.2420	0.2269	0.2753	0.9466	0.8876	0.9417	0.8944
D_{14}f	0.2327	0.2347	0.2285	0.3041	1.0086	0.9818	1.0127	1.0047
D_{14}	0.2302	0.2347	0.2285	0.3066	1.0194	0.9926	1.0223	1.0076
D_{15}f	0.2095	0.2266	0.2286	0.3352	1.0814	1.0912	1.0828	1.1161
D_{15}	0.2073	0.2264	0.2286	0.3377	1.0922	1.1028	1.1035	1.1235

Table S2b. The theoretical fractions of main peaks and isotope peaks in the total amount of the substance, the theoretical (M+1)/M and (M+2)/M ratios between main peak and isotope peaks, and the calculated median (M+1)/M ratios in two tunnels of hydroxylated methylsiloxanes in aerosol samples. Calculated median ratios refer to the median ratios estimated after subtracting the contribution of the main+2 isotope peaks of the corresponding cVMS.

Siloxane	Fractions				Theoretical ratios		Calculated median ratios
	Main	Main+1	Main+2	Rest	(M+1)/M	(M+2)/M	(M+1)/M
$D_2T^{OH}f$	0.7441	0.1467	0.0921	0.0171	0.1972	0.1238	0.3750
D_2T^{OH}	0.7361	0.1531	0.0927	0.0181	0.2080	0.1260	0.2182
$D_3T^{OH}f$	0.6699	0.1809	0.1171	0.0321	0.2700	0.1748	0.4494
D_3T^{OH}	0.6628	0.1861	0.1178	0.0333	0.2808	0.1777	na
$D_4T^{OH}f$	0.6032	0.2068	0.1394	0.0506	0.3428	0.2310	0.3359
D_4T^{OH}	0.5968	0.2110	0.1401	0.0521	0.3536	0.2347	0.3609
$D_5T^{OH}f$	0.5431	0.2257	0.1589	0.0723	0.4156	0.2925	0.3247
D_5T^{OH}	0.5373	0.2291	0.1596	0.0740	0.4264	0.2970	0.2428

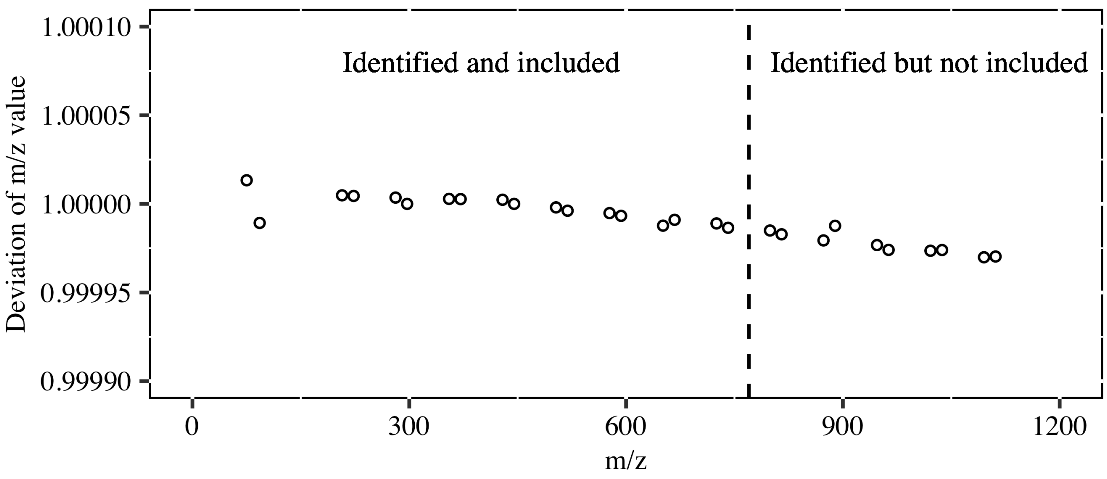

Figure S1. The deviation of the detected m/z value from the theoretical m/z value.

The deviation of the detected m/z value from the theoretical m/z value is shown Figure S1. The instrument was well calibrated in the m/z < ~800 range and this was considered a valid range. On the other hand, the detected peaks increasingly deviated from the theoretical m/z values in the m/z > 800 range, but we can still identify these peaks as methylsiloxanes based on the isotope peaks and (M+1)/M ratios. According to the PDMS depolymerization described in Figure 2f, the concentrations of cVMS products are in descending order of molecular size, which means that the peaks of large molecular products may become so low that they cannot be distinguished from blanks. Therefore, we set a cutoff point for conservative calculations of concentrations and emission factors, when methylsiloxane peaks were not significantly different from blanks, even though some methylsiloxane peaks were still detected by the PTR-MS. As a result, methylsiloxanes of D_3–D_{10} in tunnel samples were statistically different from the blank (Welch t-test, $p = 0.0033$), but methylsiloxanes of D_{11}–D_{15} were not (Welch t-test, $p = 0.3070$). In the following calculations, only D_1–D_{10} were included for concentrations and emission factors of methylsiloxanes in vehicle emissions. Furthermore, there might still be some large molecular methylsiloxanes that cannot be thermally desorbed (up to 350 °C) by our TD-PTR-MS, so this study was originally a conservative estimation due to the temperature limitation.

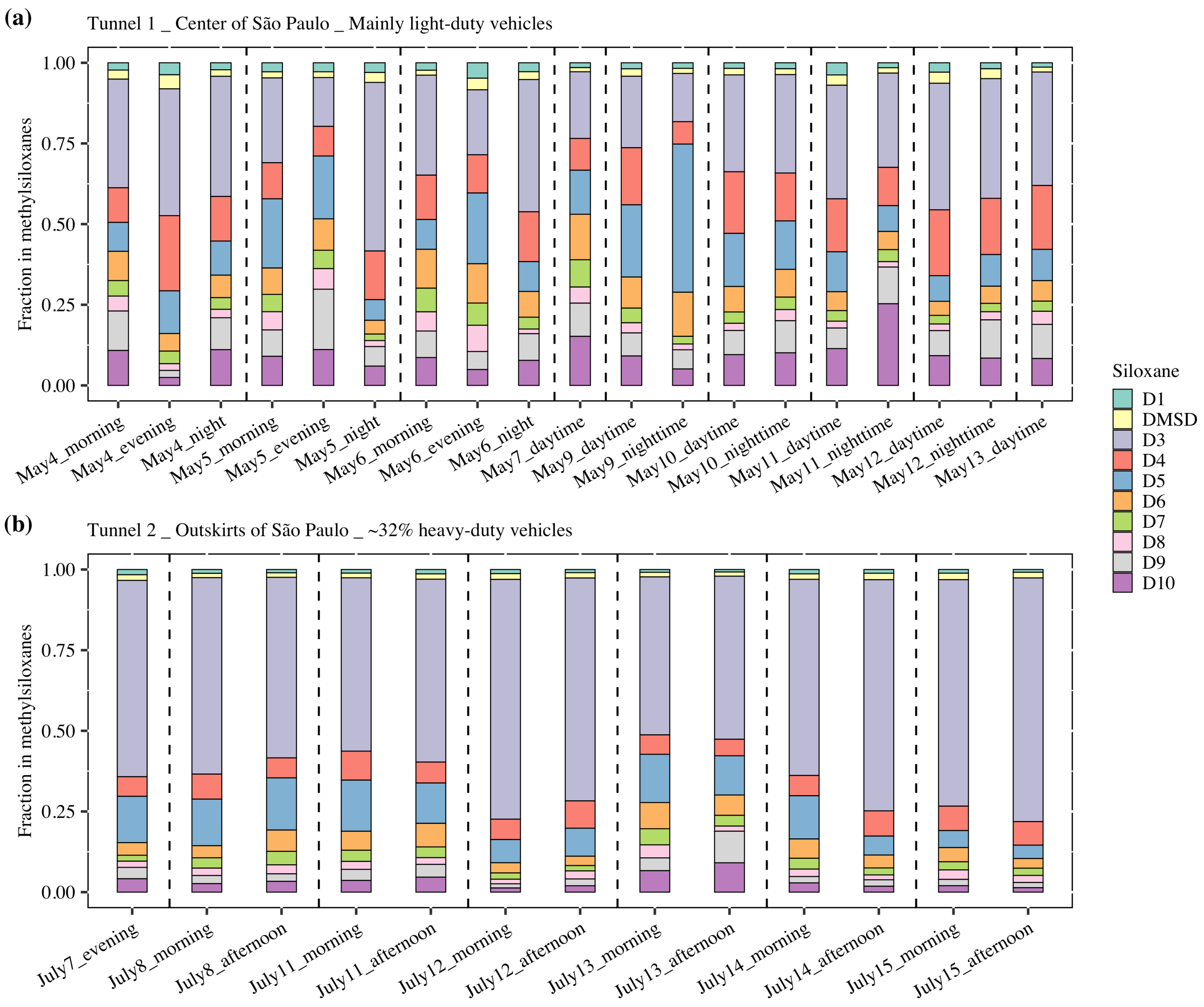

Figure S2. Fractions of D_1–D_{10} in methylsiloxanes from vehicle emissions in two tunnels in Brazil. (a) Tunnel 1 Jânio Quadros located in the center of São Paulo. (b) Tunnel 2 RodoAnel Mário Covas located on the outskirts of the city on a highway ring.

A considerable amount of D_2 (fragments of two $Si(CH_3)_2O$ units) was found in ship emissions,[1] but no D_2 was identified in mass spectra of the tunnel samples. This indicates relatively less fragmentation, which might be related to the concentrations of methylsiloxanes.

Table S3. D_1–D_{10} methylsiloxanes and C_{23}–C_{38} hydrocarbons from lubricating oils detected in the aerosol samples collected in Tunnel 1. Percentage refers to the fraction of pollutants in organic aerosols (OA) detected by the PTR-MS.

Tunnel 1	Sample ID	D_1–D_{10} methylsiloxane		C_{23}–C_{38} Hydrocarbon	
		Concentration (ng m^{-3})	Percentage (%)	Concentration (ng m^{-3})	Percentage (%)
May 4th, morning	Tunnel1_01	82.7	1.0	1190	14
May 4th, evening	Tunnel1_02	58.2	0.78	1630	22
May 4th, night	Tunnel1_03	37.7	1.1	558	16
May 5th, morning	Tunnel1_04	92.9	1.3	1180	16
May 5th, evening	Tunnel1_05	160	1.8	1380	16
May 5th, night	Tunnel1_06	86.0	1.0	1110	13
May 6th, morning	Tunnel1_08	136	1.4	1870	19
May 6th, evening	Tunnel1_09	159	1.3	1990	16
May 6th, night	Tunnel1_10	48.4	0.98	724	15
May 7th, daytime	Tunnel1_11	70.6	1.5	724	16
May 9th, daytime	Tunnel1_12	51.1	1.2	846	19
May 9th, nighttime	Tunnel1_13	73.2	1.9	530	14
May 10th, daytime	Tunnel1_14	44.6	0.90	1190	24
May 10th, nighttime	Tunnel1_15	43.9	1.1	679	18
May 11th, daytime	Tunnel1_16	49.1	0.88	1090	20
May 11th, nighttime	Tunnel1_17	104	1.5	1100	15
May 12th, daytime	Tunnel1_18	52.0	0.86	1350	22
May 12th, nighttime	Tunnel1_19	38.9	0.94	617	15
May 13th, daytime	Tunnel1_20	43.7	0.96	1100	24
Maximum		160	1.9	1990	24
Minimum		37.7	0.78	530	13
Average		75.4	1.2	1100	18
Standard deviation		39.2	0.3	420	4

Table S4. D_1–D_{10} methylsiloxanes and C_{23}–C_{38} hydrocarbons from lubricating oil detected in the aerosol samples collected in Tunnel 2. Percentage refers to the fraction of pollutants in OA detected by the PTR-MS.

Tunnel 2	Sample ID	D_1–D_{10} methylsiloxane		C_{23}–C_{38} hydrocarbons	
		Concentration (ng m^{-3})	Percentage (%)	Concentration (ng m^{-3})	Percentage (%)
Day1_evening	Tunnel2_01	174	1.0	3850	22
Day2_morning	Tunnel2_02	238	1.2	4450	21
Day2_afternoon	Tunnel2_03	191	1.1	3700	21
Day3_morning	Tunnel2_04	220	1.1	3800	19
Day3_afternoon	Tunnel2_05	197	1.1	3090	18
Day4_morning	Tunnel2_06	217	1.0	3980	18
Day4_afternoon	Tunnel2_07	207	1.1	3650	19
Day5_morning	Tunnel2_08	377	1.3	5980	21
Day5_afternoon	Tunnel2_09	209	1.4	2930	19
Day6_morning	Tunnel2_10	271	1.1	4060	17
Day6_afternoon	Tunnel2_11	214	0.98	4000	18
Day7_morning	Tunnel2_15	225	1.0	4210	19
Day7_afternoon	Tunnel2_12	233	1.0	4060	18
Maximum		377	1.4	5980	22
Minimum		174	0.98	2930	17
Average		229	1.1	3980	19
Standard deviation		51	0.1	730	2

C_{23}–C_{38} hydrocarbons from unburned lubricating oil were conservatively estimated in this study, because there should be some short-chain fragments and oxidized products due to engine combustion, but this part overlapped with the mass spectra of fuel oils, and is therefore not included in the calculation.

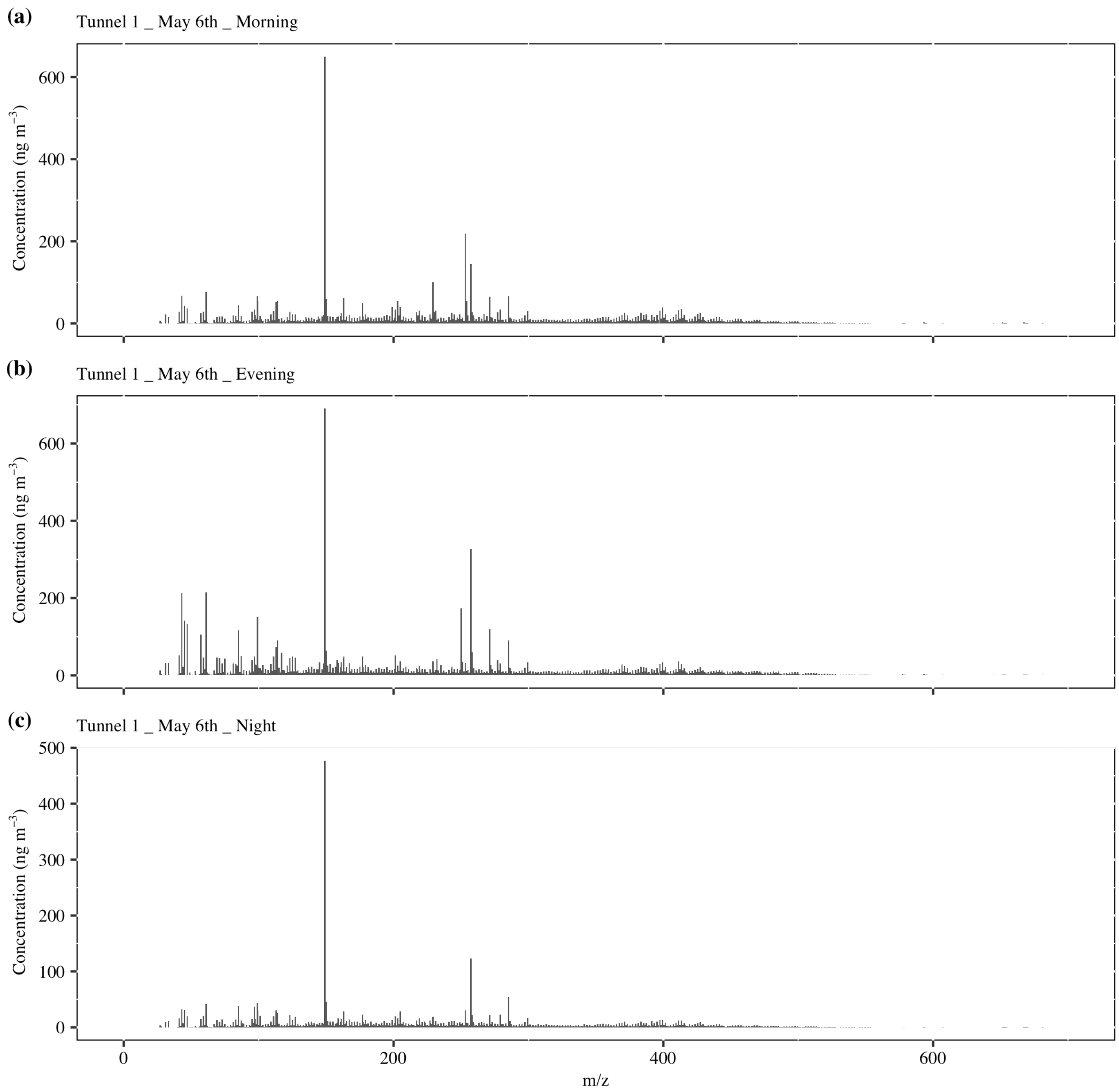

Figure S3. Full mass spectra of the aerosol samples collected in Tunnel 1, (a) morning, (b) evening, and (c) night on May 6th.

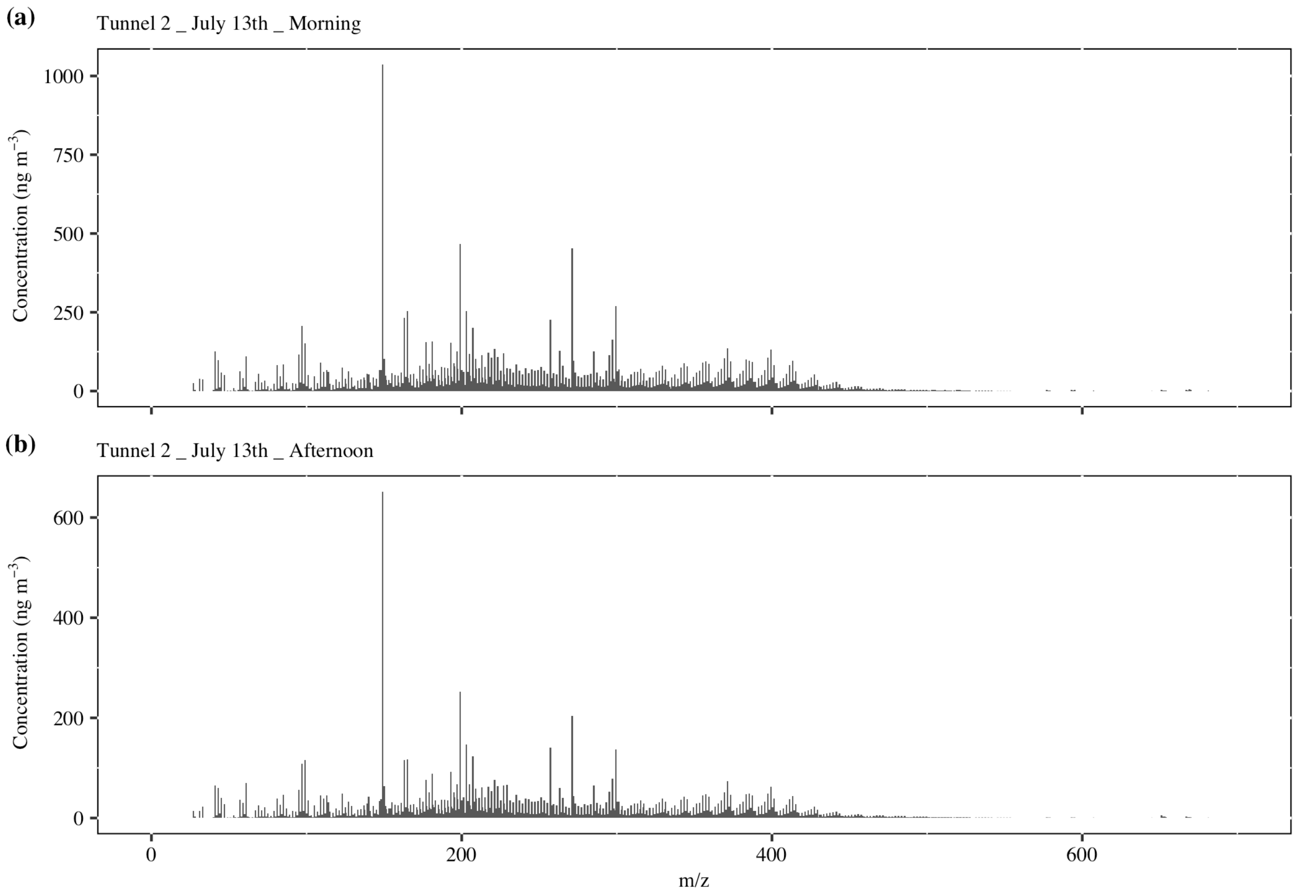

Figure S4. Full mass spectra of the aerosol samples collected in Tunnel 2, (a) morning and (b) afternoon on July 13th.

S2. Molecular size analysis of methylsiloxanes in particulate vehicle emissions

The correlation between viscosity and the degree of polymerization is important for the estimation of molecular size in this study. Therefore, the Table 1 in Mojsiewicz-Pieńkowska et al.[2] was adapted here for convenience, as shown in Table S5.

Table S5. Dependence of molecular weight and viscosity PDMS on the degree of polymerization. (adapted from Mojsiewicz-Pieńkowska et al.[2])

Degree of polymerization number n	Molecular weight [Da]	Viscosity [cSt]	Type of polymer
2	237	1	Low molecular weight
8	770	5	
15	1250	10	
25	2000	20	
50	3500	50	Middle molecular weight
80	6000	100	
125	9500	200	
160	12000	300	
190	13600	350	
400	30000	1000	
650	48000	5000	
800	60000	10000	
1230	91000	30000	High molecular weight
1500	116500	60000	
1870	139000	100000	

Table S6. Thermal desorption of commercially available PDMS standards at elevated temperature steps first in He and then in O_2, shown as percentages of total carbon.

Temperature (°C)	Atmosphere	PDMS5	PDMS10	PDMS20	PDMS50	PDMS100	PDMS1000	PDMS10000
100	He	8.3	1.7	1.8	1.4	0.6	1.6	1.8
150	He	38	13	1.9	1.1	0.4	1.3	1.5
200	He	26	24	8.8	1.0	0.3	1.1	1.3
250	He	14	22	16	3.9	1.5	1.0	1.4
300	He	5.3	14	17	10	5.4	1.3	1.7
350	He	1.4	6.6	13	12	7.3	1.8	2.0
400	He	0.3	5.0	9.9	13	9.8	2.5	2.6
450	He	0.1	2.7	5.6	10	10	3.8	3.1
500	He	0.0	1.3	4.4	8.8	12	6.2	4.6
550	He	0.0	0.8	4.1	7.4	15	16	12
600	He	0.0	0.7	3.7	5.5	9.7	26	29
650	He	0.0	0.7	3.2	4.2	4.6	12	14
850	O_2	6.1	7.7	11	22	24	26	24

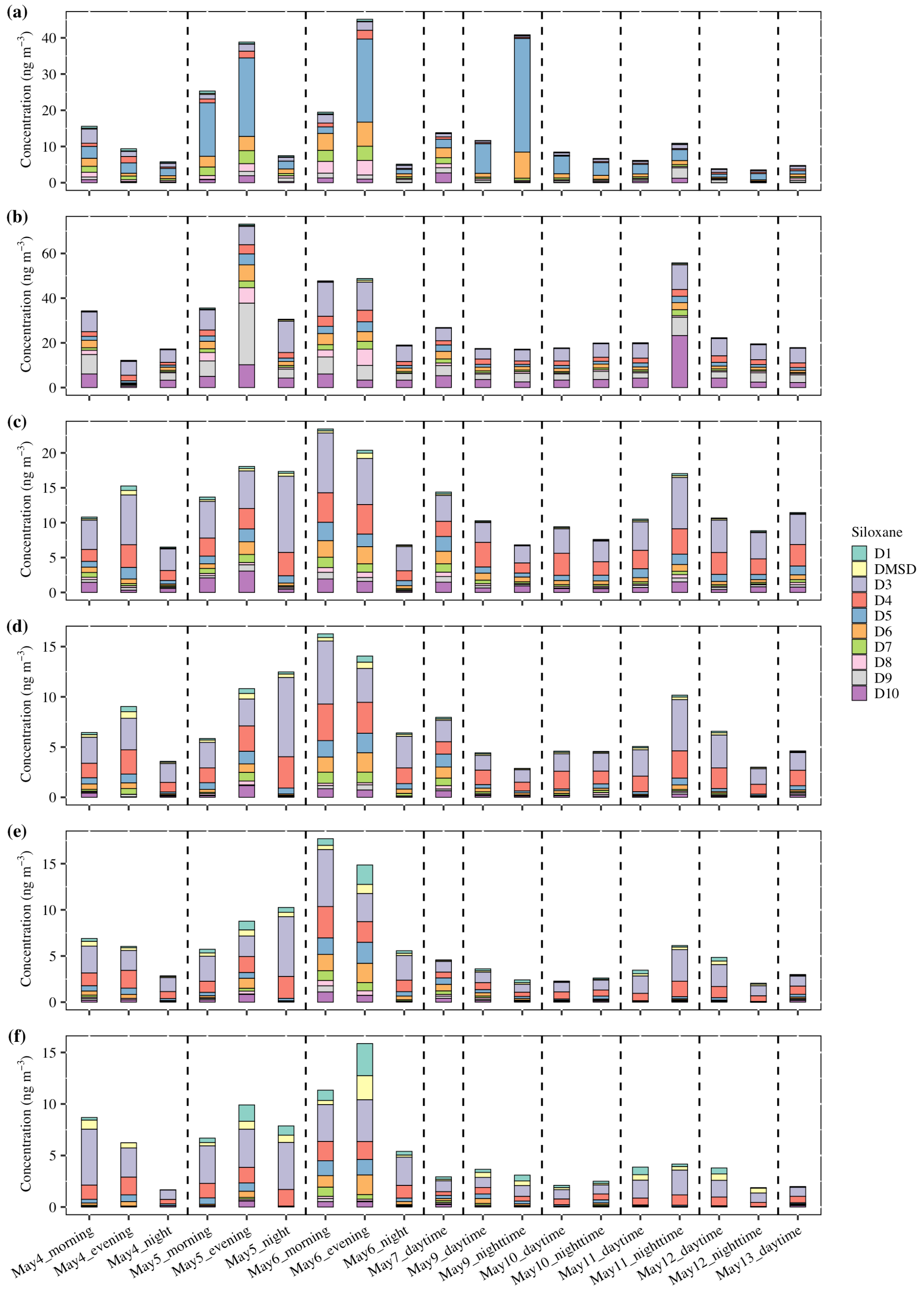

Figure S5. Concentrations of D_1–D_{10} methylsiloxanes in the aerosol samples collected in Tunnel 1, desorbed at elevated temperature steps, including (a) 100 °C, (b) 150 °C, (c) 200 °C, (d) 250 °C, (e) 300 °C, and (f) 350 °C.

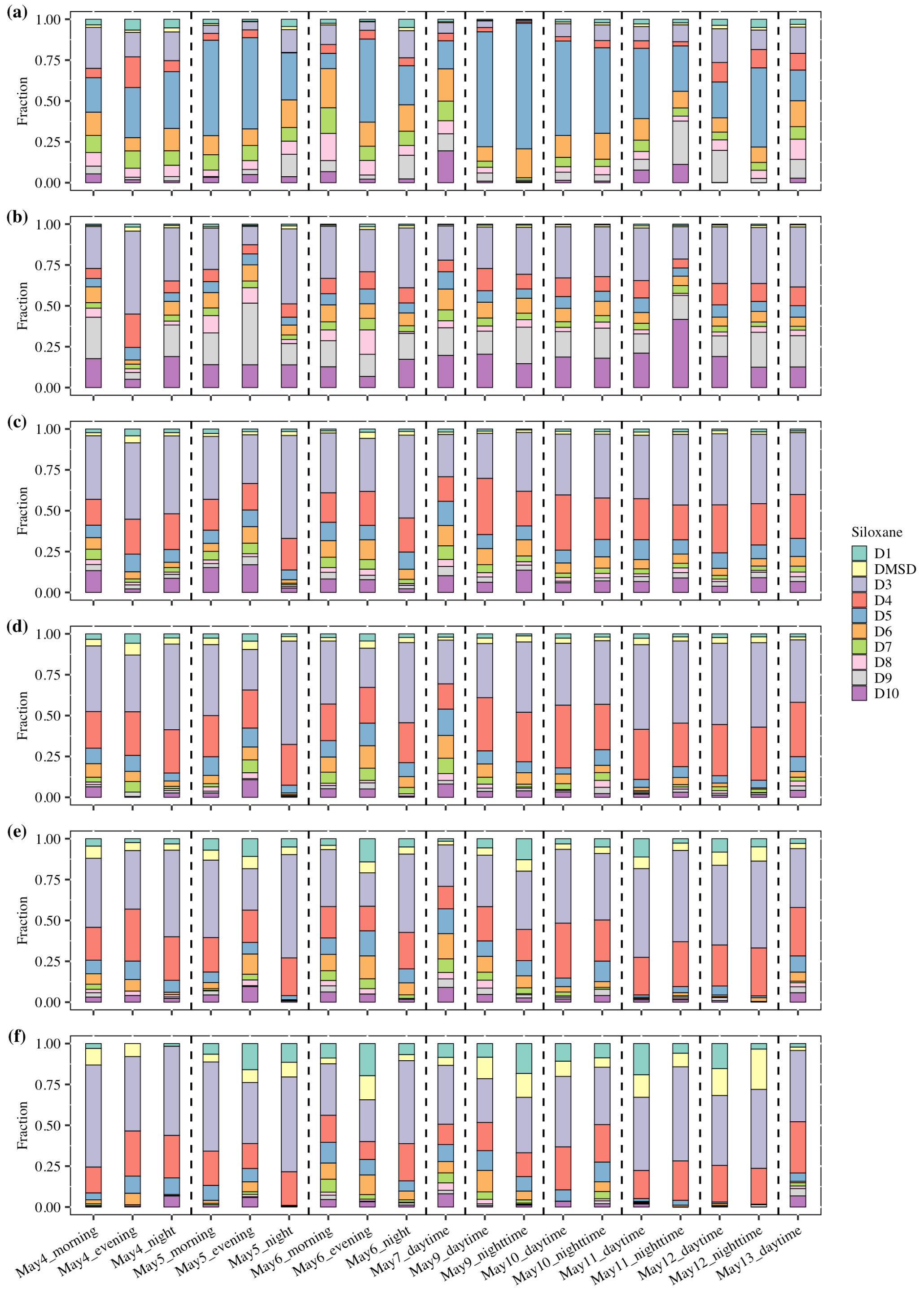

Figure S6. Fractions of D_1–D_{10} in the detected methylsiloxanes in the aerosol samples collected in Tunnel 1, desorbed at elevated temperature steps, including (a) 100 °C, (b) 150 °C, (c) 200 °C, (d) 250 °C, (e) 300 °C, and (f) 350 °C.

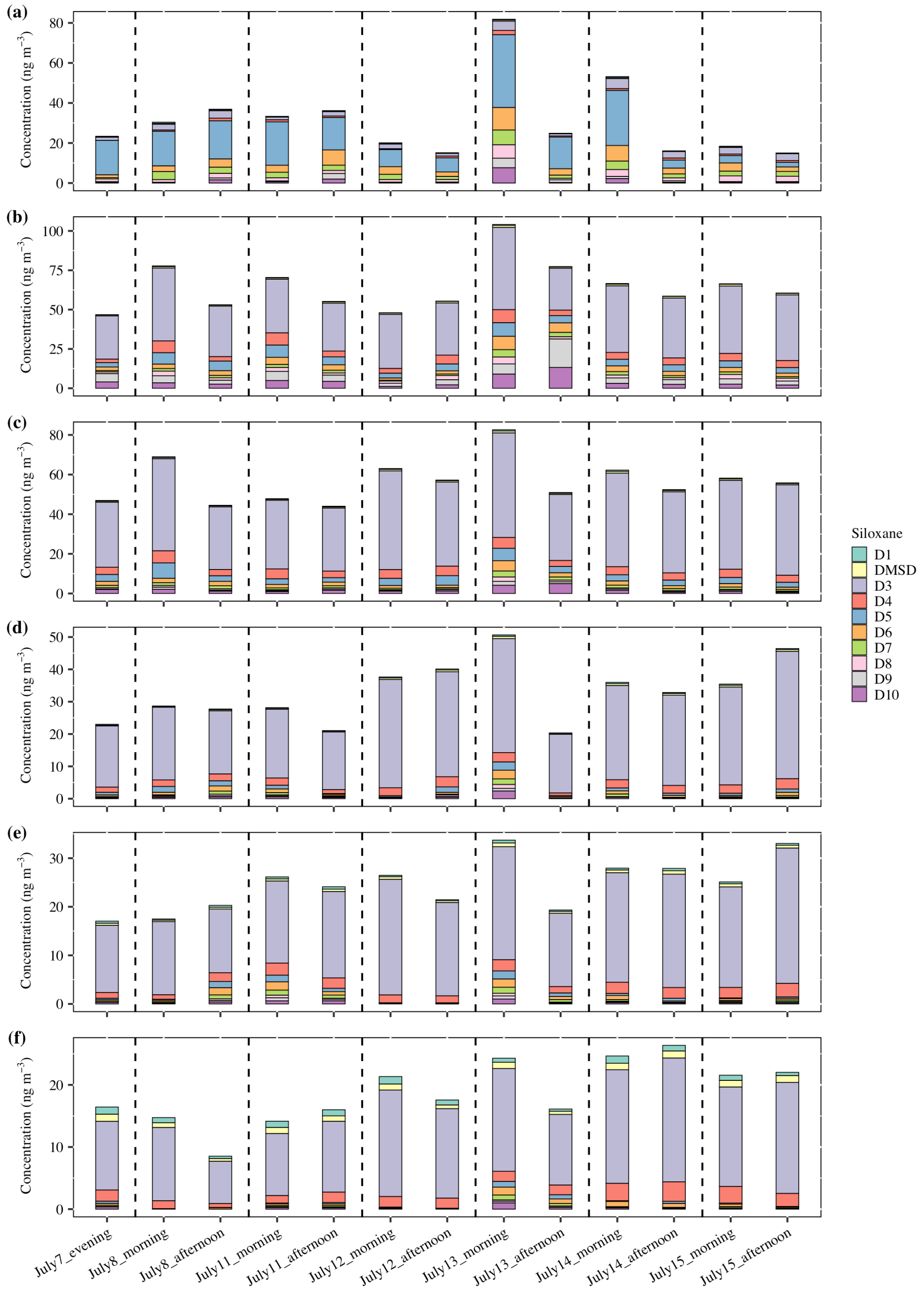

Figure S7. Concentrations of D_1–D_{10} methylsiloxanes in the aerosol samples collected in Tunnel 2, desorbed at elevated temperature steps, including (a) 100 °C, (b) 150 °C, (c) 200 °C, (d) 250 °C, (e) 300 °C, and (f) 350 °C.

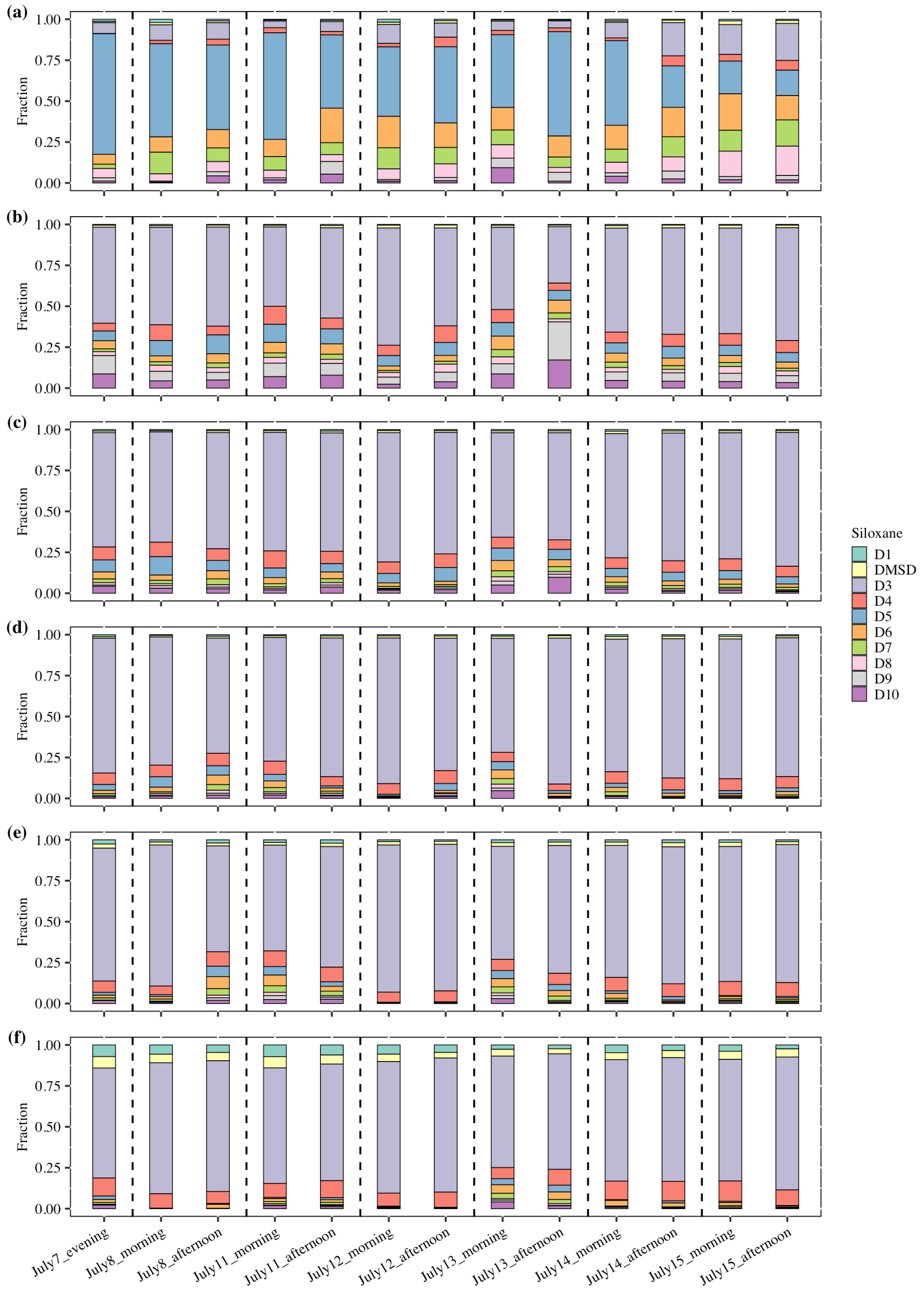

Figure S8. Fractions of D_1–D_{10} in the detected methylsiloxanes in the aerosol samples collected in Tunnel 2, desorbed at elevated temperature steps, including (a) 100 °C, (b) 150 °C, (c) 200 °C, (d) 250 °C, (e) 300 °C, and (f) 350 °C.

Table S7. Averaged concentrations of D_1–D_{10} methylsiloxanes in the aerosol samples collected in the tunnels, desorbed at elevated temperature steps.

Temperature (°C)	Methylsiloxanes in Tunnel 1 Concentration (ng m−3)	Methylsiloxanes in Tunnel 2 Concentration (ng m^{-3})
100	15.3	31.8
150	30.0	66.1
200	14.3	57.3
250	8.09	33.8
300	6.54	25.3
350	6.01	18.9

The molecular sizes of some fragments and original PDMS can be much larger than the detection range of various ordinary mass spectrometry (e.g., GC-MS). Therefore, taking the advantage of thermal desorption and depolymerization, TD-PTR-MS shows certain advantages in the detection of methylsiloxanes with large molecular sizes.

S3. The relationships between methylsiloxanes, hydrocarbons from lubricating oils, and organic aerosols.

The relationships between D_1–D_{10} methylsiloxanes, C_{23}–C_{38} hydrocarbons, and organic aerosols (OA) are shown in Figure S9. The positive intercept in Figure S9a (and Figure 3e) indicated other potential sources of methylsiloxanes, which may be related to non-combustion vehicle emissions.

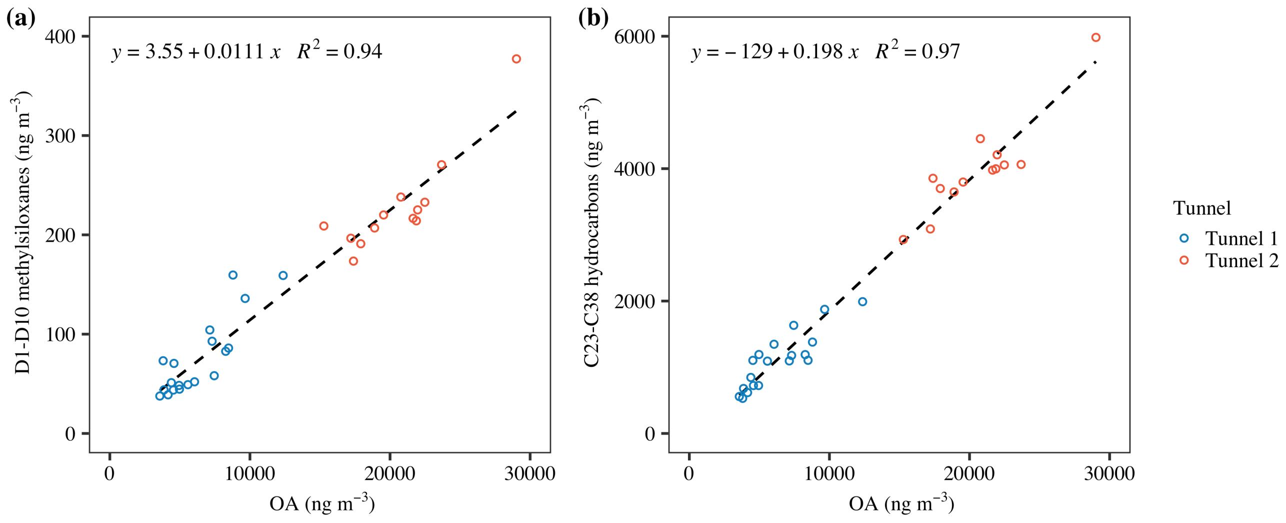

Figure S9. The relationships between organic aerosols and (a) D_1–D_{10} methylsiloxanes, (b) C_{23}–C_{38} hydrocarbons. The black dashed line is the linear fit.

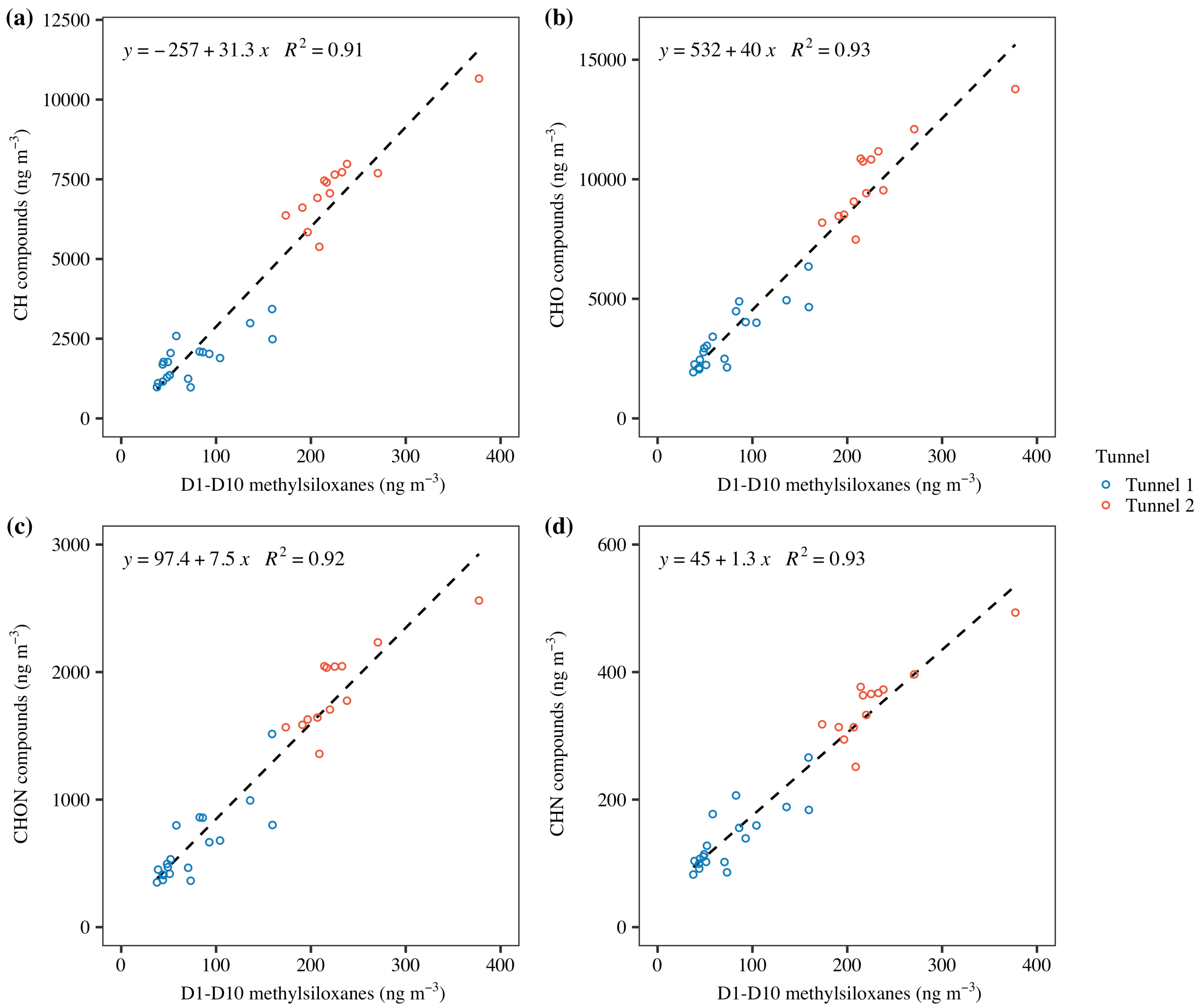

Figure S10. The relationships between D_1–D_{10} methylsiloxanes and organic compounds, including (a) CH compounds, (b) CHO compounds, (c) CHON compounds, and (d) CHN compounds. The black dashed line is the linear fit.

The desorbed organic compounds CH, CHO, CHON, CHN, methylsiloxanes, and others accounted for 32%, 51%, 10%, 2.0%, 1.1%, and 4.5% of the organic aerosol (OA) on average, respectively. Since C_{23}–C_{38} hydrocarbons from lubricating oils accounted for 18% of OA on average, the unburned hydrocarbons were mainly large molecules from lubricating oils rather than fuel oils. The concentration relationships between methylsiloxanes and CH, CHO, CHON, CHN compounds are shown Figure S10. The R^2 values

were very close, between 0.91–0.93. This suggested that the methylsiloxanes came from vehicle emissions, but did not point to a specific composition or combustion process.

Furthermore, when investigating the concentration relationship between methylsiloxanes and each desorbed molecule, 565 molecules out of 1116 had R^2 values higher than 0.8, 114 molecules had R^2 values higher than 0.9, and only 7 molecules had an R^2 value higher than 0.93. This again indicated that methylsiloxanes emissions did not originate from a specific source or process, but rather from fuel oils and lubricating oils.

S4 Calculation of emission factors

S4.1 Emission factors in two different units

The emission factor is calculated by equation (1).[3–5]

$$\mathrm{EF_P} = \left(\frac{\Delta[\mathrm{P}]}{\Delta[\mathrm{CO_2}] + \Delta[\mathrm{CO}]}\right) \times w_c \times 10^3 \tag{1}$$

EF_P refers to the emission factor of pollutant P (mg kg^{-1}), which means mg of pollutants emitted per kg of fuel burned. Δ[P] refers to the concentration of the pollutant (ng m^{-3}), and this study focuses on the methylsiloxanes and hydrocarbons from lubricating oils. Δ[CO_2] and Δ[CO] refer to concentrations of CO_2 and CO emitted by vehicles in units of μg C m^{-3}. In tunnel studies, Δ[CO_2] and Δ[CO] are calculated by the difference between the concentrations inside and outside of the tunnel, using 12 g C mol^{-1} for both CO_2 or CO. w_c is the carbon mass fraction of the fuel (g C per g fuel), with w_G = 0.757 g C g^{-1} for gasohol and w_D = 0.818 g C g^{-1} for diesel.

With the tunnel size and other information, EF_p* can also be given in units of mg pollutant emitted per kilometer km per vehicle (mg km^{-1} $vehicle^{-1}$):

$$\mathrm{EF_p^*} = \mathrm{EF_P} \times \left[\left(\Delta[\mathrm{CO_2}] \times \frac{44}{12}\right) \times \frac{S \times v \times t}{\mathrm{N} \times l} \times \frac{\rho}{c}\right] \times 10^{-6} \tag{2}$$

Equation (2) can be separated into equations (S1–S2).

$$\mathrm{EF_p^*} = \mathrm{EF_P} \times R \times 10^{-3} \tag{S1}$$

$$R = \left(\Delta[\mathrm{CO_2}] \times \frac{44}{12}\right) \times \frac{S \times v \times t}{\mathrm{N} \times l} \times \frac{\rho}{c} \times 10^{-3} \tag{S2}$$

R refers to the fuel consumption rate of vehicle (g km^{-1} $vehicle^{-1}$), which means g of fuel consumed per km per vehicle. *S* refers to the cross-sectional area of the tunnel (m^2). *v* refers to the velocity of the air wind measured inside the tunnel (m s^{-1}). *t* refers to the time interval (s). N refers to the number of vehicles passing the tunnel at the time t. *l* refers to the tunnel length (m). *ρ* refers to the fuel density (g L^{-1}). *c* refers

to the carbon intensity of the fuel (g CO_2 L^{-1}). The factor 44/12 refers to the unit conversion from g C to g CO_2.

S4.2 Light-duty vehicles (LDV)

The vehicle type in Tunnel 1 is dominated by light-duty vehicles (gasohol). Therefore, the emission factors of light-duty vehicles can be estimated based on the samples from Tunnel 1, referred as EF_{P_LDV} and $EF^*_{P\_LDV}$. The parameters of Tunnel 1 and property parameters of gasohol can be applied into equations (1) and (2), shown as equations (S3) and (S4).

$$\mathrm{EF_{P\_LDV}} = \left(\frac{\Delta[\mathrm{P}]_G}{\Delta[\mathrm{CO_2}]_G + \Delta[\mathrm{CO}]_G}\right) \times w_c \times 10^3 \tag{S3}$$

$$\mathrm{EF^*_{P\_LDV}} = \mathrm{EF_{P\_LDV}} \times \left[\left(\Delta[\mathrm{CO_2}]_G \times \frac{44}{12}\right) \times \frac{S \times v \times t}{\mathrm{N} \times l} \times \frac{\rho}{c}\right] \times 10^{-6} \tag{S4}$$

S4.3 Heavy-duty vehicles (HDV)

Previous tunnel studies have shown that emissions from light-duty and heavy-duty vehicles have similar CO emission rates per kilometer.[3,6] The CO emissions from heavy-duty vehicles (diesel) can therefore be estimated according to the equation (S5).

$$\Delta[\mathrm{CO}]_D = f_D \times \Delta[\mathrm{CO}] \tag{S5}$$

$\Delta[CO]_D$ refers to CO concentrations of emissions from heavy-duty vehicles in unit (μg C m^{-3}). f_D is the number fraction of heavy-duty vehicles, $f_D = N_D / (N_G + N_D)$. N_D and N_G refer to the numbers of heavy-duty and light-duty vehicles passing the tunnel at the time t, respectively.

The CO_2 emissions from heavy-duty vehicles can be estimated according to the equation (S6).[4–7]

$$\frac{\Delta[\mathrm{CO_2}]_D}{\Delta[\mathrm{CO_2}]} = \frac{f_D U_D \rho_D w_D}{f_D U_D \rho_D w_D + (1 - f_D) U_G \rho_G w_G} \tag{S6}$$

$$R = U \times \rho \times 10^{-2} \tag{S7}$$

$\Delta[CO_2]_D$ refers to CO_2 concentrations of emissions from heavy-duty vehicles in unit (μg C m^{-3}). f_D is the number fraction of heavy-duty vehicles. U refers to the fuel consumption rate in unit (L 100^{-1} km^{-1} $vehicle^{-1}$). ρ refers to the fuel density (g L^{-1}). w_c is the carbon mass fraction of the fuel (g C g^{-1}). $U \times \rho$ can be converted into another fuel consumption rate R in unit (g km^{-1} $vehicle^{-1}$) by equation (S7). Instead of $U \times \rho$, the average fuel consumption rate R (g km^{-1} $vehicle^{-1}$) was used in calculation in this study, with R_D and R_G in Table S9.

As the emissions from Tunnel 1 were dominated by light-duty vehicles (gasohol), the ratio between $(\Delta[P]_G/\Delta[CO]_G)_{Tunnel\_1}$ can be used as the ratio for light-duty vehicles $\Delta[P]_G/\Delta[CO]_G$. The emissions of heavy-duty vehicles in Tunnel 2 can be estimated by equation (S8).

$$\Delta[\mathrm{P}]_D = \Delta[\mathrm{P}] - \Delta[\mathrm{CO}] \times (1 - f_D) \times \left(\frac{\Delta[\mathrm{P}]_G}{\Delta[\mathrm{CO}]_G}\right)_{\mathrm{Tunnel\_1}} \tag{S8}$$

Where $\Delta[P]_D$ is the concentration of a pollutant emitted from heavy-duty vehicles (ng m^{-3}). $(1 - f_D)$ refers to the fraction of $\Delta[CO]$ from light-duty vehicles. $(\Delta[P]_G/\Delta[CO]_G)_{Tunnel\_1}$ is the estimated ratio of pollutant over CO concentrations of light-duty vehicles based on results of Tunnel 1.

With $\Delta[CO]_D$, $\Delta[CO_2]_D$, and $\Delta[P]_D$ obtained from equations (S3, S4, S6), the emission factors of heavy-duty vehicles can be calculated based on equations (1) and (2), referred as EF_{P_HDV} and $EF^*_{P\_HDV}$. In calculations, the parameters of Tunnel 2 and property parameters of diesel should be used in equations (1) and (2), shown as equations (S9) and (S10).

$$\mathrm{EF_{P\_HDV}} = \left(\frac{\Delta[\mathrm{P}]_D}{\Delta[\mathrm{CO_2}]_D + \Delta[\mathrm{CO}]_D}\right) \times w_c \times 10^3 \tag{S9}$$

$$\mathrm{EF^*_{P\_HDV}} = \mathrm{EF_{P\_HDV}} \times \left[\left(\Delta[\mathrm{CO_2}]_D \times \frac{44}{12}\right) \times \frac{S \times v \times t}{\mathrm{N} \times l} \times \frac{\rho}{c}\right] \times 10^{-6} \tag{S10}$$

S4.4 Conditions, parameters, and detailed results

Table S8. Parameters of the tunnels.

	Tunnel 1	Tunnel 2
Cross-sectional area of the tunnel	$S_{Tunnel\_1} = 80.6\ m^2$	$S_{Tunnel\_2} = 100.5\ m^2$
Velocity of the air wind in the tunnel	$v_{Tunnel\_1} = 6.1\ m\ s^{-1}$	$v_{Tunnel\_2} = 4.9\ m\ s^{-1}$
Tunnel length	$l_{Tunnel\_1} = 850\ m$	$l_{Tunnel\_2} = 1150\ m$

Table S9. Properties of fuel oils.

Fuel type	Gasohol	Diesel
Vehicle type	Light-duty	Heavy-duty
Carbon mass fraction of the fuel	$w_G = 0.757$ g C g^{-1}	$w_D = 0.818$ g C g^{-1}
Fuel density	$\rho_G = 765$ g L^{-1}	$\rho_D = 854$ g L^{-1}
Carbon intensity of the fuel	$c_G = 2331$ g CO_2 L^{-1}	$c_D = 2772$ g CO_2 L^{-1}
Fuel consumption rate	$R_G = 75$ g km^{-1} $vehicle^{-1}$	$R_D = 251$ g km^{-1} $vehicle^{-1}$
Fuel consumption rate	$U_G = 9.8$ L 100^{-1} km^{-1} $vehicle^{-1}$	$U_D = 29.4$ L 100^{-1} km^{-1} $vehicle^{-1}$

Table S10. Vehicle numbers, sampling conditions, and pollutant concentrations in Tunnel 1.

Sample ID	LDV	HDV	Time (h)	CO_2 inside (ppm)	CO inside (ppm)	CO_2 outside (ppm)	CO outside (ppm)	Siloxane (ng m^{-3})	Hydrocarbons (ng m^{-3})
	N_{LDV}	N_{HDV}	t					$\Delta[P]_{MS}$	$\Delta[P]_{HC}$
Tunnel1_01	13920	29	6	513.6	5.10	403.2	1.33	82.7	1190
Tunnel1_02	12856	34	3	526.3	6.15	401.1	1.14	58.2	1630
Tunnel1_03	13584	36	12	456.0	2.66	416.2	1.09	37.7	558
Tunnel1_04	14759	49	5	513.6	5.47	403.2	1.33	92.9	1180
Tunnel1_05	12252	6	3	526.3	7.06	401.1	1.14	160	1380
Tunnel1_06	13538	18	12	456.0	4.42	416.2	1.09	86	1110
Tunnel1_08	13338	19	6	513.6	7.25	403.2	1.33	136	1870
Tunnel1_09	12660	6	3	526.3	7.37	401.1	1.14	159	1990
Tunnel1_10	12363	43	12	456.0	3.58	405.1	1.09	48.4	724
Tunnel1_11	24510	272	12	510.5	3.47	400.4	1.24	70.6	724
Tunnel1_12	25067	387	12	511.0	5.04	394.5	1.26	51.1	846
Tunnel1_13	11546	36	12	425.9	2.05	390.5	0.75	73.2	530
Tunnel1_14	31258	79	12	498.9	5.41	405.4	1.45	44.6	1190
Tunnel1_15	13113	111	12	437.5	2.50	392.5	0.49	43.9	679
Tunnel1_16	36288	1223	12	507.3	5.45	395.7	0.73	49.1	1090
Tunnel1_17	13274	86	12	488.1	3.32	458.5	1.88	104	1100
Tunnel1_18	32800	283	11	512.2	5.79	393.9	1.29	52	1350
Tunnel1_19	14209	35	13	436.3	2.40	393.2	0.76	38.9	617
Tunnel1_20	27162	87	12	510.0	5.35	403.1	1.35	43.7	1100
Average								75.4	1100
SD								39.2	420

The unit of CO_2 and CO concentrations were converted from ppm into μg C m^{-3}, using 12 g C mol^{-1} for both CO_2 or CO.

Table S11. Emission factors of light-duty vehicles of methylsiloxanes and hydrocarbons from lubricating oils.

Sample ID	EF_{MS_LDV} mg kg^{-1}	EF_{HC_LDV} mg kg^{-1}	R_G g km^{-1} vehicle^{-1}	$EF^*_{MS\_LDV}$ mg km^{-1} vehicle^{-1}	$EF^*_{HC\_LDV}$ mg km^{-1} vehicle^{-1}
Tunnel1_01	1.02	14.7	63.7	0.0653	0.940
Tunnel1_02	0.631	17.7	39.1	0.0247	0.693
Tunnel1_03	1.29	19.1	47.1	0.0606	0.897
Tunnel1_04	1.15	14.5	50.0	0.0573	0.727
Tunnel1_05	1.72	14.9	41.1	0.0707	0.612
Tunnel1_06	2.82	36.2	47.3	0.133	1.71
Tunnel1_08	1.65	22.8	66.6	0.110	1.52
Tunnel1_09	1.71	21.4	39.8	0.0681	0.852
Tunnel1_10	1.28	19.2	66.1	0.0846	1.27
Tunnel1_11	0.888	9.10	71.6	0.0635	0.651
Tunnel1_12	0.601	9.94	73.7	0.0443	0.733
Tunnel1_13	2.82	20.4	49.2	0.139	1.01
Tunnel1_14	0.647	17.3	48.1	0.0311	0.831
Tunnel1_15	1.32	20.4	54.8	0.0724	1.12
Tunnel1_16	0.597	13.3	47.9	0.0286	0.636
Tunnel1_17	4.74	49.9	35.7	0.169	1.78
Tunnel1_18	0.599	15.5	52.8	0.0316	0.819
Tunnel1_19	1.23	19.5	52.8	0.0649	1.03
Tunnel1_20	0.557	14.1	63.2	0.0352	0.889
Average	1.16	16.7	54.6	0.0619	0.895
SD	0.59	3.9	11.0	0.0300	0.240

Sample Tunnel1_06 and Tunnel1_17 are outliers, which was caused by abnormal $\Delta[CO_2]$, so these two samples were not included for the calculation of the average of emission factors and further calculation of heavy-duty vehicles in Tunnel 2. Sample Tunnel1_13 is also an outlier of especially high methylsiloxanes emissions, so this sample was not included for the emission factor calculation of methylsiloxanes.

Table S12. Vehicle numbers, sampling conditions, and pollutant concentrations in Tunnel 2.

Sample ID	LDV<br>N_{LDV}	HDV<br>N_{HDV}	Time (h)<br>t	CO_2 inside (ppm)	CO inside (ppm)	CO_2 outside (ppm)	CO outside (ppm)	Siloxane (ng m^{-3})<br>$\Delta[P]_{MS}$	Hydrocarbons (ng m^{-3})<br>$\Delta[P]_{HC}$
Tunnel2_01	10497	4189	6	671.5	3.90	405.7	0.78	174	3850
Tunnel2_02	8406	4401	6	681.7	3.49	415.5	0.83	238	4450
Tunnel2_03	14432	5171	6	661.9	3.91	416.8	0.74	191	3700
Tunnel2_04	7675	3960	5	678.1	4.56	417.1	1.49	220	3800
Tunnel2_05	10807	4865	7	689.4	3.91	416.8	0.91	197	3090
Tunnel2_06	9836	5030	6	746.5	5.35	417.6	2.09	217	3980
Tunnel2_07	12860	5441	6	679.1	3.85	416.8	0.96	207	3650
Tunnel2_08	10585	5426	6	696.1	4.79	417.6	1.01	377	5980
Tunnel2_09	11739	5311	7	678.8	4.62	416.8	1.04	209	2930
Tunnel2_10	10751	5386	6	754.1	6.68	417.6	2.43	271	4060
Tunnel2_11	11795	5112	6	683.5	4.31	416.8	1.15	214	4000
Tunnel2_15	10400	5354	6	694.4	4.83	417.6	1.15	225	4210
Tunnel2_12	14351	5142	5	689.0	3.62	416.8	1.00	233	4060
Average								229	3980
SD								51	730

Table S13. Emission factors of heavy-duty vehicles of methylsiloxanes and hydrocarbons from lubricating oils.

Sample ID	EF_{MS_HDV} mg kg^{-1}	EF_{HC_HDV} mg kg^{-1}	R_D g km^{-1} vehicle^{-1}	$EF*_{MS\_HDV}$ mg km^{-1} vehicle^{-1}	$EF*_{HC\_HDV}$ mg km^{-1} vehicle^{-1}
Tunnel2_01	1.16	29.3	219	0.255	6.43
Tunnel2_02	1.69	32.9	230	0.387	7.57
Tunnel2_03	1.47	31.3	157	0.231	4.90
Tunnel2_04	1.52	27.3	208	0.315	5.68
Tunnel2_05	1.31	21.2	236	0.310	5.00
Tunnel2_06	1.17	22.7	247	0.288	5.61
Tunnel2_07	1.50	27.8	170	0.255	4.73
Tunnel2_08	2.61	42.1	194	0.506	8.16
Tunnel2_09	1.40	19.5	208	0.291	4.05
Tunnel2_10	1.41	21.5	234	0.331	5.04
Tunnel2_11	1.49	29.7	186	0.277	5.52
Tunnel2_15	1.40	26.9	195	0.274	5.25
Tunnel2_12	1.81	34.2	146	0.264	4.99
Average	1.53	28.2	202	0.306	5.61
SD	0.37	6.2	31	0.072	1.15

S5 Engine lubrication of vehicles and ships

In the engine cylinder lubrication, the lubricating oil is mainly used to reduce heat and friction between the pistons and the liners, to wash away the generated soot and combustion residues, and to reduce corrosive wear by neutralizing the acid of the combustion products.

In vehicle engines and marine engines for small vessels, cylinder lubrication is a part of the engine lubrication system. The lubricating oil is sprayed onto the piston rings from below, and due to the protective isolation of the piston rings and oil wiper rings, the lubricating oil cannot splash into the combustion chamber. A small amount of lubricating oil creates an oil film between the piston and cylinder wall.

In marine engines for large vessels, the cylinder lubrication system is usually independent from the main lubricating oil system. Lubricating oil is fed into the engine cylinder directly through oil holes, which are in the cylinder wall in conjunction with the vertical movement of the piston. An adequate amount of oil film will be maintained between the piston and cylinder wall for boundary lubrication. Waste lubricating oil is discharged from a drain hole at the bottom of the cylinder.

The differences in lubrication method and engine size leads to a higher degree of lubricating oil combustion in the engine of large marine vessels. In addition, the high speed of the pistons in a car engine means shorter combustion time, while the piston speed of a marine engine is usually much slower, which means sufficient time for oxidation.

References

(1) Yao, P.; Chianese, E.; Kairys, N.; Holzinger, R.; Materić, D.; Sirignano, C.; Riccio, A.; Ni, H.; Huang, R.-J.; Dusek, U. A Large Contribution of Methylsiloxanes to Particulate Matter from Ship Emissions. *Environ. Int.* **2022**, *165*, 107324. https://doi.org/10.1016/j.envint.2022.107324.

(2) Mojsiewicz-Pieńkowska, K. Size Exclusion Chromatography with Evaporative Light Scattering Detection as a Method for Speciation Analysis of Polydimethylsiloxanes. III. Identification and Determination of Dimeticone and Simeticone in Pharmaceutical Formulations. *J. Pharm. Biomed. Anal.* **2012**, *58* (1), 200–207. https://doi.org/10.1016/j.jpba.2011.09.003.

(3) Marr, L. C.; Kirchstetter, T. W.; Harley, R. A.; Miguel, A. H.; Hering, S. V.; Hammond, S. K. Characterization of Polycyclic Aromatic Hydrocarbons in Motor Vehicle Fuels and Exhaust Emissions. *Environ. Sci. Technol.* **1999**, *33* (18), 3091–3099. https://doi.org/10.1021/es981227l.

(4) Pérez-Martínez, P. J.; Miranda, R. M.; Nogueira, T.; Guardani, M. L.; Fornaro, A.; Ynoue, R.; Andrade, M. F. Emission Factors of Air Pollutants from Vehicles Measured inside Road Tunnels in São Paulo: Case Study Comparison. *Int. J. Environ. Sci. Technol.* **2014**, *11* (8), 2155–2168. https://doi.org/10.1007/s13762-014-0562-7.

(5) Oyama, B. S.; Andrade, M. D. F.; Herckes, P.; Dusek, U.; Röckmann, T.; Holzinger, R. Chemical Characterization of Organic Particulate Matter from On-Road Traffic in São Paulo, Brazil. *Atmos. Chem. Phys.* **2016**, *16* (22), 14397–14408. https://doi.org/10.5194/acp-16-14397-2016.

(6) Pierson, W. R.; Gertler, A. W.; Robinson, N. F.; Sagebiel, J. C.; Zielinska, B.; Bishop, G. A.; Stedman, D. H.; Zweidinger, R. B.; Ray, W. D. Real-World Automotive Emissions—Summary of Studies in the Fort McHenry and Tuscarora Mountain Tunnels. *Atmos. Environ.* **1996**, *30* (12), 2233–2256. https://doi.org/10.1016/1352-2310(95)00276-6.

(7) W. Kirchstetter, T.; Harley, R. A.; Kreisberg, N. M.; Stolzenburg, M. R.; Hering, S. V. On-Road

Measurement of Fine Particle and Nitrogen Oxide Emissions from Light- and Heavy-Duty Motor Vehicles. *Atmos. Environ.* **1999**, *33* (18), 2955–2968. https://doi.org/10.1016/S1352-2310(99)00089-8.
